# Facet selectivity in gold binding peptides: exploiting interfacial water structure†
†Electronic supplementary information (ESI) available: Text, figures and tables (including a detailed description of the computational methods and analysis; a description of the three metadynamics re-weighting schemes; a full description of analyses associated with the composition of polycrystalline gold surfaces, and structural analyses of the peptide; results of secondary structural, structure clustering and residue binding analyses obtained using the ‘Time Period’ and ‘Bonomi’ re-weighting methods; and, a discussion of the convergence of the metadynamics simulations); a collation of *in vacuo* GolP-CHARMM binding energies of amino acid analogues adsorbed on the three facets; side-chain/surface distances in the peptide adsorbed state for four residues across the three facets; typical configurations of the peptide adsorbed on the three facets superimposed against the 3-D water density. See DOI: 10.1039/c5sc00399g



**DOI:** 10.1039/c5sc00399g

**Published:** 2015-06-23

**Authors:** Louise B. Wright, J. Pablo Palafox-Hernandez, P. Mark Rodger, Stefano Corni, Tiffany R. Walsh

**Affiliations:** a Dept. of Chemistry , University of Warwick , Coventry , CV4 7AL , UK; b Institute for Frontier Materials , Deakin University , Geelong , 3216 , VIC , Australia . Email: tiffany.walsh@deakin.edu.au; c Centre for Scientific Computing , University of Warwick , Coventry , CV4 7AL , UK . Email: p.m.rodger@warwick.ac.uk; d Centro S3 CNR Istituto Nanoscienze , Modena , Italy . Email: stefano.corni@nano.cnr.it

## Abstract

Peptide sequences that can discriminate between gold facets under aqueous conditions offer a promising route to control the growth and organisation of biomimetically-synthesised gold nanoparticles. Knowledge of the interplay between sequence, conformations and interfacial properties is essential for predictable manipulation of these biointerfaces, but the structural connections between a given peptide sequence and its binding affinity remain unclear, impeding practical advances in the field. These structural insights, at atomic-scale resolution, are not easily accessed with experimental approaches, but can be delivered *via* molecular simulation. A current unmet challenge lies in forging links between predicted adsorption free energies derived from enhanced sampling simulations with the conformational ensemble of the peptide and the water structure at the surface. To meet this challenge, here we use an *in situ* combination of Replica Exchange with Solute Tempering with Metadynamics simulations to predict the adsorption free energy of a gold-binding peptide sequence, AuBP1, at the aqueous Au(111), Au(100)(1 × 1) and Au(100)(5 × 1) interfaces. We find adsorption to the Au(111) surface is stronger than to Au(100), irrespective of the reconstruction status of the latter. Our predicted free energies agree with experiment, and correlate with trends in interfacial water structuring. For gold, surface hydration is predicted as a chief determining factor in peptide–surface recognition. Our findings can be used to suggest how shaped seed-nanocrystals of Au, in partnership with AuBP1, could be used to control AuNP nanoparticle morphology.

## Abstract 2

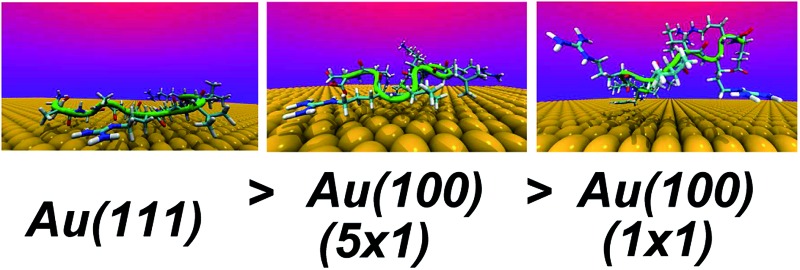
We demonstrate that surface hydration is a key factor in dictating the free energy of non-covalent peptide-materials recognition.

## Introduction

The ability to control the size, shape and self-organised assembly of noble metal nanoparticles (NPs) is pivotal to realizing their full potential in many nanotechnological applications. For instance, if it were possible to construct a 3-D array comprising NPs of such well-controlled size and shape, with precise positional and orientational control, this would deliver an unprecedented tunability of the plasmonic absorption frequency. To date, synthetic strategies for size- and shape-selective production of NPs *under aqueous conditions* reported in the literature can be broadly grouped into two classes: seed mediated growth (see *e.g.* Jiang *et al.* ^1^); and those aided by the presence of additives: ions,^2,3^ surfactants^3–6^ and peptides.^7–13^ Biomimetic protocols, harnessing the selectivity of biomolecules, such as proteins and peptides, offer great promise in particular. Not only do they offer a ‘green’ synthetic route for the production of NPs, but they can also be used to rationally direct the organisation of NPs into well-defined nanostructures.^14–22^

Here we focus on the aqueous peptide–gold interface; of interest to a range of fields including biomimetic materials synthesis^13,23–28^ and medicine.^29–37^ For gold, the only currently-available ‘additive-mediated’ synthetic strategies (in liquid media) are those that produce anisotropic AuNPs with rod-like dimensions—*e.g.* see Danger *et al.* ^5^ Unlike platinum^7,10,11,38^ and palladium,^2^ analogous procedures that can exert fine control over *spherical* AuNP morphology and facet composition in liquid media are lacking. Following the precedent set by Forbes *et al.* ^7^ for platinum, and further refined by Chiu *et al.*,^10^ one strategy to realise the goal of shape- and size-control of AuNP's in aqueous solution would be to identify peptides able to discriminate between different crystallographic planes of gold. To date, however, biocombinatorial selection of gold-binding sequences has not focused on facet selectivity, (*i.e.* previous selection studies used polycrystalline gold,^23,39,40^ colloidal gold particles^41,42^ or gold powder^43^ as targets). Only the GBP1 peptide sequence^44^ was selected against a single crystallographic plane, namely Au(111).

To fully exploit the opportunities presented by peptide-materials selective binding, rational, knowledge-based design of facet-selective peptide sequences derived from detailed knowledge of the fundamental mechanisms that drive gold adsorption is essential. To enable this, a deeper molecular-level understanding of the phenomena that drives peptide adsorption, capable of delivering discrimination between crystallographic planes of gold, is required. However, despite recent advances^45–48^ it remains challenging to probe biointerfacial adsorption experimentally. In addition, experimental peptide–gold binding studies have not, to date, reported characterisation that *contrasts* adsorption at different gold facets.^23,40,49–51^ To complement experiments, molecular simulation can, in principle, be used to obtain such information, at the required atomistic level of detail.

Herein, we report use of advanced molecular dynamics (MD) simulations to quantify and characterise peptide adsorption at the aqueous Au(111), Au(100)(1 × 1) and Au(100)(5 × 1) interfaces, and thus elucidate the factors that govern facet-specific peptide adsorption at aqueous Au interfaces. We have focused on the gold-binding peptide sequence AuBP1;^23^ this sequence, WAGAKRLVLRRE, was originally biocombinatorially-selected against polycrystalline gold targets. The adsorption of this peptide sequence at aqueous polycrystalline gold interfaces been extensively characterised by experiment.^23,50^ Prior to considering the effects of NP features (such as edges and vertices) on peptide–gold facet-specific adsorption, here, we have investigated and elucidated the binding characteristics of AuBP1 adsorbed to the planar Au crystallographic surfaces that are lowest in energy, under ideal, defect-free conditions. Not only are the Au(111) and Au(100) surfaces commonly-featured AuNP facets,^52^ but the relative length scales of AuNPs (typically grown under aqueous conditions) and combinatorially-selected materials-binding peptides make modeling of adsorption using planar interfaces an acceptable approximation to experiment.^53^

Despite previous reports that have used molecular simulation to contrast peptide adsorption at the aqueous Au(111) and Au(100)(1 × 1) surfaces,^54–58^ a number of concerns remain. First, genuine adsorption *free* energies have never been calculated for peptides adsorbed at the bare aqueous Au interface. Previous studies have reported the enthalpic contribution to adsorption with an approximate estimate of the entropy.^54,55^ However, the conformational entropy contributions of the peptide and the liberation entropy of the gold-hydration layer are, in general, non-negligible, and they may affect binding differently at different interfaces.^59,60^ Second, previous simulation studies that compared peptide adsorption at Au(111) and Au(100) only considered the native form of Au(100).^54^ However, it is well known that under conditions relevant to biomolecule adsorption, the *planar* Au(100) surface is predominantly present in its reconstructed form,^61^ Au(100)-*hex* ^62–64^ (modeled here as Au(100)(5 × 1)). Furthermore, there is no knowledge of the reconstructed status of the Au(100) facet on the surface of a AuNP under aqueous conditions.^65–69^ Thus it is imperative to predict peptide adsorption onto both native and reconstructed forms of the Au(100) surface. Our previous investigation of amino acid adsorption at the aqueous Au(100)(1 × 1) and Au(100)(5 × 1) interfaces was the first to suggest that differences in water structuring at these interfaces could lead to differential peptide–surface binding affinities for these two (100) surfaces. Third, the polarisation of gold atoms, induced by the presence of a charged or polar adsorbate, was not incorporated within the force-field^70–72^ used in these previous studies.^54–58^ Gold polarisation is a contribution to the interaction energy thought to be significant at metallic surfaces, such as Au(100)(1 × 1), where the epitaxial match between adsorbate and gold is poor.^55^

**Fig. 1:**
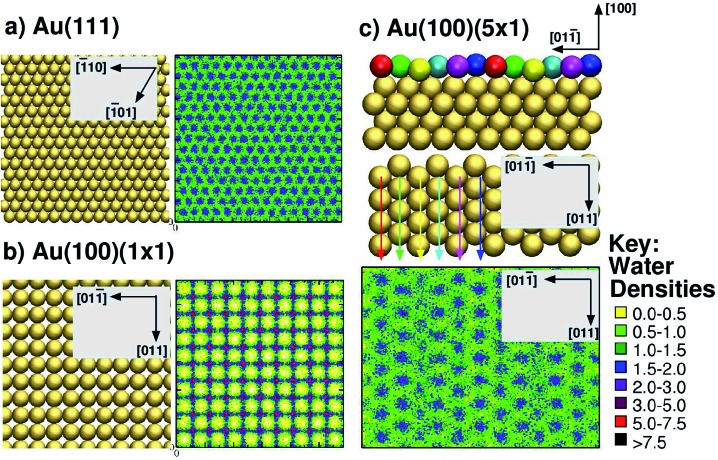
Summary of interfacial water structuring at the three gold facets. (a and b) View from above of the surface and lateral water densities (kg m^–3^) of the first interfacial water layer (see text for definition) for Au(111) and Au(100)(1 × 1),^73^ (c) side view (top of sub-panel) and view from above (centre of sub-panel) of Au(100)(5 × 1), and the lateral water density (kg m^–3^)^74^ of the first interfacial water layer (bottom of sub-panel). The differently coloured spheres indicate the six unique binding sites on the Au(100)(5 × 1) surface and are not related to the colour scheme used for the water densities (see key, right). Arrow colors are consistent with the coloring of the unique surface sites, and indicate how each row of unique surface sites is situated in the surface plane (when rotating from side view to a view from above).

To address these issues, here, we used the GolP-CHARMM suite of force-fields (FFs)^73,74^ to model all peptide–gold interactions presented herein. This FF incorporates parameters specifically derived for modeling protein adsorption at all three relevant interfaces: the Au(111), native Au(100) and reconstructed Au(100) facets. The GolP-CHARMM FF also includes terms to account for the polarisation of surface gold atoms. In addition, here we aim to probe the role of interfacial water structuring^74^ in determining the preferential adsorption of a peptide to one facet over another. The relevance of interfacial water structure has been previously suggested *via* qualitative analysis of simulations modeling amino acid adsorption at the three aqueous planar gold interfaces, Au(111),^73,75^ Au(100)(1 × 1) and Au(100)(5 × 1).^74^ Water structuring has also been identified as very influential for determining peptide adsorption to oxide surfaces.^76–79^ It is expedient, therefore, to investigate if, and how, this influence extends to aqueous metallic interfaces as well. Our previous results suggested that biomolecule–gold binding was mediated by direct (*i.e.* not water-mediated) contact with surface atoms only in the case of the Au(111) and Au(100)(5 × 1) facets, which share a similar *in-plane* quasi-hexagonal atomic arrangement. In contrast, adsorption to Au(100)(1 × 1), which features a square arrangement of surface gold atoms, was mediated by a strongly-structured layer of interfacial water, in a similar manner to that reported for aqueous metal-oxide interfaces, such as titania.^76,77^ For convenience, the lateral mass density profiles of the first water layer for each of the three interfaces are summarised in Fig. 1. In these, we show the lateral density of the first interfacial solvent layer, as defined by the range of water–surface distances up to and including the lowest point of the first trough in the vertical density profile.

The large size and complexity of solvated biointerfacial systems demand that the conformation of the adsorbate is adequately sampled during the simulation. Approaches based on the use of tens to hundreds of standard MD simulations may yield insights on the dynamical adsorption behaviour of peptides.^80^ However in view of comparing thermodynamics results such as the free energy of adsorption with experiments, accurately sampling a Boltzmann-weighted ensemble of conformations is decisive. To this aim, a well-established strategy^13,50,77,81–84^ is to use Replica Exchange Molecular Dynamics (REMD)-based methods.^85^ A REMD-based approach alone is typically not sufficient to allow a reasonable estimate of the peptide–surface adsorption free energy. On the other hand, a widely-used approach to calculate free energy differences is metadynamics,^86^ in which the sampling of the potential energy landscape along a pre-defined set (typically one or two) of collective variables (CVs), is driven *via* the addition of a history-dependent bias potential. In the case of peptide adsorption at interfaces, the distance between the peptide center-of-mass (com) and the surface represents such a CV. Nonetheless, the use of metadynamics alone cannot guarantee adequate sampling of the peptide in conformational space, orthogonal to the chosen CV, without access to impractically-long simulation times. We note here the existence of alternatives to the use of metadynamics, such as umbrella sampling and steered molecular dynamics. However, in a recent study, Deighan and Pfaendtner^84^ reported that umbrella sampling could not provide reliable free energy profiles in the case of surface adsorption of a flexible peptide. Earlier examples of the application of umbrella sampling to biomolecular adsorption did not consider a substantial conformational change upon adsorption (see for example the studies of Battle *et al.* ^87^ and Nimlos *et al.* ^88^). A steered MD approach reported by Mijajlovic *et al.* ^89^ for surface adsorption of a penta-peptide showed similar challenges in free energy profile convergence.

Following the precedent set by Latour and co-workers who used replica-exchange with a biased potential,^90^ here we have combined^77^ a Hamiltonian-based REMD method, Replica Exchange with Solute Tempering (REST)^91–94^ simulations, with metadynamics^95^ (metaD) simulations, to fully explore the adsorption of AuBP1 at each of the three aqueous gold interfaces. We obtained the free energy profile as function of the peptide–surface distance, from which the adsorption free energy was extracted and compared with experimental values. Moreover, these simulations provide access to the Boltzmann-weighted conformational ensemble for AuBP1 in solution and adsorbed at the different aqueous interfaces, allowing us to elucidate the underlying structural factors that govern the differences in adsorption free energy. Finally, we have used our findings to propose and discuss possible impacts of Au-facet differential peptide binding on AuNP synthesis in the presence of the AuBP1 peptide.

## Methodology

All simulations of AuBP1 (ref. 23) reported here were carried out using the software package GROMACS 4.5.5,^96^ incorporating a customised version of PLUMED 1.3.^97^ Four different systems were considered: the isolated peptide in solution, and, the peptide adsorbed at the aqueous Au(111), Au(100)(1 × 1) and Au(100)(5 × 1) interfaces. The Au(100)(5 × 1) surface used here is well established as a reasonable structural model of the Au(100)-*hex* reconstruction.^74^ The in-solution simulation comprised the AuBP1 peptide solvated by 6605 TIP3P water molecules in a cubic simulation cell of length 58.28 Å. Orthorhombic cells of dimensions 58.60 × 60.90 × 67.60 Å^3^, 58.60 × 58.60 × 67.60 Å^3^ and 58.60 × 58.60 × 76.51 Å^3^ were used for the interfacial simulations carried out at the Au(111), Au(100)(1 × 1) and Au(100)(5 × 1) surfaces, respectively. These comprised the AuBP1 peptide, a gold slab (5/5/9 layers thick for the three surfaces, respectively) and 6605/6540/6355 TIP3P water molecules. The protonation state of the peptide corresponded with pH 7 solution conditions, with three Cl^–^ counterions added to balance the charge. In all simulations, CHARMM22*^98,99^ was chosen to model the peptide, while the modified TIP3P^100,101^ potential was used to represent water. Peptide–gold interactions at each of the different aqueous gold interfaces were described by GolP-CHARMM.^73,74^

REST^92,94^ simulations were set up in an analogous manner to our previous work with only the peptide and counterions belonging to the ‘solute’ group.^50,81^ Briefly, a total of sixteen replicas were used to span an ‘effective temperature’ range of 300–433 K. Each replica was initially populated with AuBP1 present in a different conformation; peptide structures were constructed by hand and featured common folded backbone secondary structural motifs. All REST simulations featured the same set of starting AuBP1 conformations; with respect to both the internal-peptide and peptide–gold (orientation with respect to the surface and distance from the surface) arrangements. The CV chosen for the interfacial REST-metaD runs was the position of the *com* of the peptide in the *z* dimension (normal to the gold surface). Three REST-metaD simulations were performed, one for each aqueous gold interface. Each run was 100 ns in duration, making a total of 1.6 μs of dynamics per facet. In addition, two REST MD simulations were carried out for comparative purposes only; one of the peptide in solution (*i.e.* without the surface present), and, one with the peptide adsorbed at the Au(111) interface. Each was of 15 ns duration. Full details of all aspects of the simulations, their post-processing, and their analysis can be found in ESI† sections ‘Computational Methods’ and ‘Analysis’.

For convenience, we briefly summarise the ‘Average weight’ scheme used here to remove the added bias from our REST-metaD trajectories. In this approach all structures with a given peptide *com*–surface distance, *s*, are weighted equally, according to eqn (1).

1 *W*(*t*) = exp(–*G*(*s*(*t*),*t*_f_)/*kT*)/*N*(*s*)

where *W*(*t*) is the weight given to a frame sampled at time *t*, *G*(*s*,*t*_f_) the symmetrised free energy profile of the system as determined at the end (*t* = *t*_f_) of the simulation (Fig. S2†), and *N*(*s*) is the total number of frames for which the peptide *com*–gold distance was *s*. The scheme is conceptually similar to that of Branduardi *et al.* ^102^ For the interfacial simulations, analysis was carried out on a latter portion of the REST-metaD trajectory, after what we determined to be an equilibration period. The length of this equilibration period (70 ns for Au(111), 50 ns for Au(100)(1 × 1), and 50 ns for Au(100)(5 × 1)) was systematically assigned in each case by histogramming the biased CV over increasing time intervals (see Fig. S3†). Further details are provided in the ESI† section ‘Metadynamics Re-weighting Schemes’.

## Results and discussion

### Surface binding strength

**Fig. 2:**
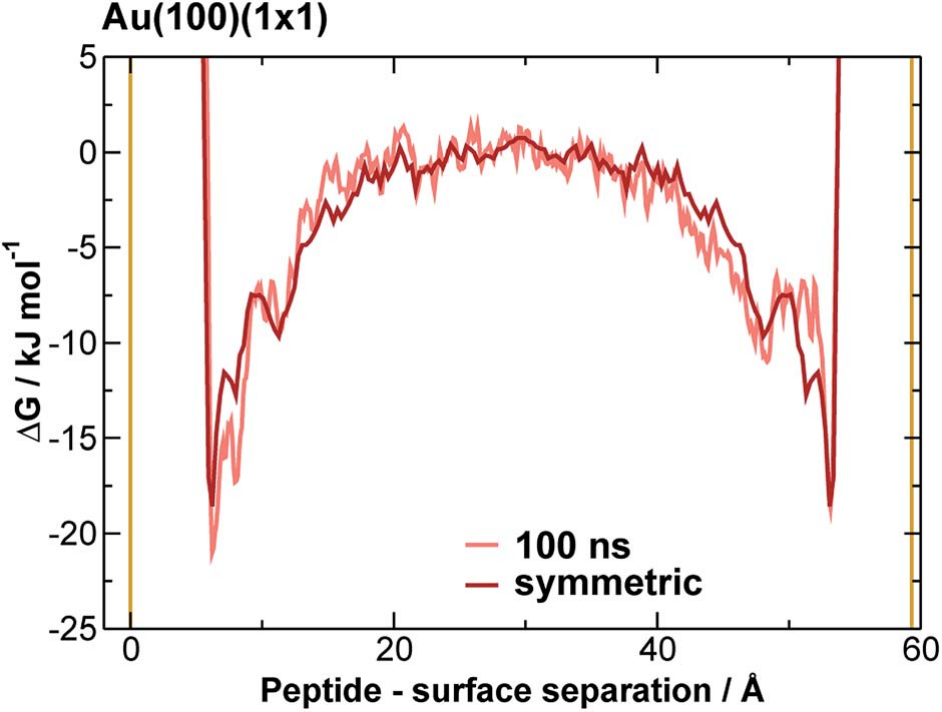
Symmetrised final free energy profile of AuBP1 adsorption at the aqueous Au(100)(1 × 1) interface. Solid yellow lines indicate the positions of the top of the gold slab, and the underside of the its periodic image along the direction perpendicular to the slab.

We extracted AuBP1 adsorption free energies *via* construction of symmetrised free energy profiles (see ESI† section ‘Methods: Free energy extraction and error analysis’), following Schneider and Colombi-Ciacchi.^77^ We show an exemplar symmetrised free energy profile in Fig. 2, and summarise these adsorption free energies for the three Au facets in Table 1. Our data in Table 1 demonstrate distinct facet-dependent differences in the *free* energy of peptide–gold adsorption. Specifically, the trend in binding affinities was clear, with Au(111) > Au(100)(5 × 1) > Au(100)(1 × 1), where the associated differences in binding free energies were predicted to be much greater than the calculated uncertainties. We therefore conclude that aqueous AuBP1 adsorption is significantly stronger at the Au(111) interface than at the Au(100) interface *irrespective* of the reconstructed status of the latter interface.

**Table 1:** Predicted free energy of adsorption of AuBP1 at the aqueous Au(111), Au(100)(1 × 1) and Au(100)(5 × 1) interfaces

	Free energy/kJ mol^–1^
Au(111)	–51.8 ± 18.1
Au(100)(1 × 1)	–10.3 ± 1.5
Au(100)(5 × 1)	–21.3 ± 4.2

This potentially could have consequences for using peptides to mediate shape-selective AuNP synthesis. Specifically, since our calculations suggest such a relatively strong affinity of AuBP1 for Au(111), we predict that AuNPs grown from cubo-octahedral seeds (small AuNPs featuring only {111} and {100} facets) in the presence of AuBP1 would possess a square bipyramidal morphology (*i.e.* the resulting AuNP would feature only {111} facets). It is noted that experimental work has shown that aqueous AuNP synthesis without a seed NP, in the presence of AuBP1, is not shape selective.^28^

As a verification of our simulations, we compared our predicted binding affinities with experimental values. There are two independently-reported experimental measurements of the adsorption free energy of AuBP1 at the aqueous polycrystalline gold interface: the first, by Hnilova *et al.*, used surface plasmon resonance spectroscopy (–37.4 ± 0.8 kJ mol^–1^)^23^ and the second, more recently reported by Tang *et al.*, from quartz crystal microbalance measurements (–37.6 ± 0.9 kJ mol^–1^).^50^ While the exact composition (in terms of the relative proportion of different facets) of the polycrystalline Au surfaces probed experimentally is unknown, it is reasonable to suppose that these strongly featured Au(111) and Au(100), the two crystallographic planes of gold that are lowest in energy. In the limit of single-molecule/Au-surface adsorption, such as that modelled here, binding to the most favorable adsorption sites, namely the Au(111) facets of polycrystalline gold, should dominate. Hence, we can approximate Δ*G*_polycryst_ ≈ Δ*G*_Au(111)_ = –51.8 ± 18.1 kJ mol^–1^. To probe the case of the opposite extreme (that of surface saturation, where binding to Au(100) facets would become relevant) we estimated the relative proportion of (111) and (100) facets on the polycrystalline Au surface, using published data for the relative surface energies of the aqueous Au(111) and Au(100) interfaces^103,104^ (see ESI† section ‘Analysis: Polycrystalline Surfaces’). By combining these published data with our predictions for the facet-specific free energy of adsorption, we can estimate that Δ*G*_polycryst_ = –37.9 ± 12.1 kJ mol^–1^ if the Au(100) surface is present in its native form, and Δ*G*_polycryst_ = –40.4 ± 11.5 kJ mol^–1^ if instead the Au(100) surface is present in the Au(100)(5 × 1) form. We propose that the regime under which the experimental binding free energies were measured most likely falls between the two extremes discussed above (that of single molecule adsorption and surface saturation) because in each experimental study, the binding data were well modelled by a Langmuir isotherm.^50^ In particular, the study reported by Tang *et al.* took special care to demonstrate that their experimental *aqueous interfacial* system did not correspond with multiple layers of adsorbed peptide.

Taking all of the above considerations into account, there is excellent agreement between experiment and our predicted binding affinities. Deviations from the experimental values might be expected due to the approximations inherent to our model. Specifically, the presence of surface defects and grain boundaries, likely to be present in the experiments, were not accounted for in our computational model; nor were contributions from adsorption to other, higher-energy crystallographic planes of Au; deviation from atomistically-flat surfaces in experiment (*i.e.* surface roughness), which may impair concomitant interactions of more than one residue, were not considered; and, the neglect of the presence of multiple peptide chains in the interfacial overlayer. Insufficient sampling of ‘in-solution’ states (where the peptide is located in the region of bulk water, midway between the gold slab and it nearest periodic image), as may be inferred by the time evolution of the free energy profiles (see Fig. 2 and S11 and S12 of the ESI†), might also contribute. However, accurate determination of the free energy of adsorption of AuBP1 onto different specific crystallographic planes of gold under aqueous conditions is not crucial in this work, as long as the relative affinity order for the three surfaces is robust with respect the free energy error bars. Here, we sought only to estimate binding affinities, and to link these to their corresponding structural properties. Analysis and discussion of the limitations of the REST-metaD approach is given in the ESI† section ‘Convergence of Metadynamics’.

In terms of comparison with previous modeling work, a stronger affinity of peptides for Au(111) than Au(100) has been reported,^54–57^ although these simulation studies only considered the *native* forms of the two aqueous gold interfaces, and did not report the genuine free energy of binding. Interestingly, our calculations confirm our earlier hypothesis^73,74^ that the adsorption of peptides to the reconstructed form of the Au(100) surface is stronger than to the native surface, under aqueous conditions. Therefore, while our findings are in agreement with current opinion in the literature, our study highlights a number of additional notable differences.

First, our findings predict that the nature of the Au(100) facets featured by a AuNP, namely native or reconstructed, will influence the thermodynamic competition in peptide adsorption between the Au(100) and Au(111) facets. In the limit of planar interfaces, the Au(100)-*hex* structure is the energetically most favorable reconstruction under aqueous conditions relevant to biomolecule adsorption.^61^

Second, our prediction of a distinct binding preference of AuBP1 between Au(111) and Au(100)(5 × 1) is in marked contrast with the ‘soft epitaxy’ mechanism of peptide–gold adsorption proposed by Heinz *et al.* ^54^ A soft epitaxy model would predict comparable adsorption at the Au(111) and Au(100)(5 × 1) interfaces, due to similarities in the spatial arrangement of surface atoms. Instead, we provide evidence to support an alternative hypothesis, that of the structure of interfacial water playing a critical role in governing peptide–gold binding, as detailed in the following. In previous work^74^ we reported a predicted trend of Au(111) < Au(100)(5 × 1) < Au(100)(1 × 1) for the density (per unit surface area) of water within the first adsorbed layer at the three aqueous interfaces. This trend is completely anti-correlated with the binding affinities of AuBP1 calculated here (Table 1), suggesting that competition between the peptide and water for direct adsorption to a gold surface is one contributing factor to our calculated facet selectivity. The first layer of adsorbed water at the bare aqueous Au(100)(1 × 1) interface is highly ordered, located specifically atop gold atom sites;^73^ in comparison, the distribution above the Au(111) and Au(100)(5 × 1) interfaces is more diffuse^73,74^ (see Fig. 1). Similar findings have been recently reported for the aqueous Pt(111) and Pt(100)(1 × 1) interfaces.^105^

**Fig. 3:**
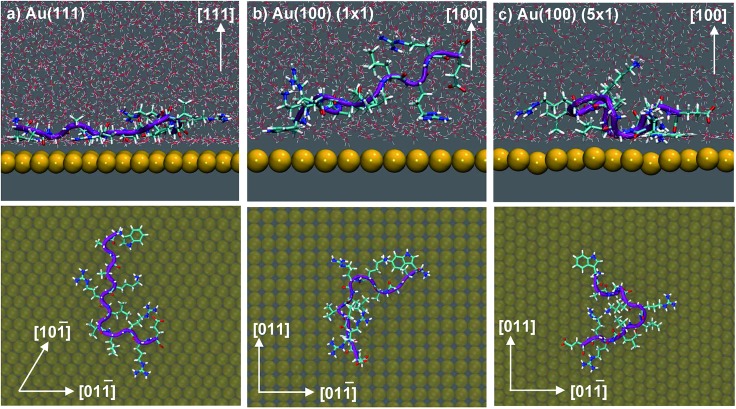
Snapshots depicting the side view (top) and view from above (bottom) of AuBP1 adsorbed at the aqueous (a) Au(111), (b) Au(100)(1 × 1) and (c) Au(100)(5 × 1) interfaces. Gold atoms are shown in yellow, hydrogen in white, oxygen in red, nitrogen in blue and carbon in cyan. Water molecules were omitted for clarity in the plan view images.

Our previously-published data for *in vacuo* adsorption of biomolecular fragments on Au(111), Au(100)(1 × 1) and Au(100)(5 × 1) surfaces^73,74,106^ underscore the influence of the interfacial solvation on the three facets. We provide a collation of these data in Table S4 of the ESI.† These GolP-CHARMM *in vacuo* adsorption data broadly demonstrate that most molecules in this set do not bind preferentially to Au(111) (defined as a difference in binding energy of 2 kJ mol^–1^ or more), but instead have comparable or stronger affinity for the Au(100) surfaces. These *in vacuo* adsorption data defy the trend shown by our binding affinities to the aqueous Au facets, and suggest that the structuring of the solvent at the interface influences these binding trends. In the case of the aqueous Au(100)(1 × 1) interface, we observe AuBP1 to penetrate only minimally through this first interfacial layer of water molecules, while for Au(111) and Au(100)(5 × 1), direct (*i.e.* non-water mediated) Au surface contact is more substantial (see Fig. 3). This was highlighted further when differences in peptide–surface contact between the three interfaces, in terms of the number and quality of contacts between the AuBP1 residues and the Au surface, was considered (section ‘Structural Analysis’).

### Structural analysis

To elucidate the origins of the facet-selective binding preferences of AuBP1 between the aqueous Au(111), Au(100)(1 × 1) and Au(100)(5 × 1) interfaces, we carried out detailed analyses of the Boltzmann-weighted ensemble of conformations extracted from our REST-metaD simulations. As has been indicated in previous studies,^50,76,81,107^ the overall affinity of a peptide for a surface is thought to be a complex interplay between the intrinsic binding propensity of the constituent residues in the sequence and the conformation of the sequence as a whole. In some cases, strong adsorption of materials-binding proteins and peptides is thought to be mediated by surface-induced folding.^108–110^ Therefore, our analyses were aimed at searching for surface-dependent differences in two key properties of the adsorbed peptide: the ensemble of internal peptide conformations, and, the residue–surface contact (see ESI† section ‘Analysis: Structural Analysis’).

To calculate average structural properties from our REST-metaD simulations, it was first necessary to re-weight the configurations in the trajectory to remove the effect of the bias potential; this is challenging because the bias is evolved continually during the simulation. Two schemes for doing this have been previously reported in the literature,^111,112^ but neither was found to be entirely suitable for our purposes. Instead, we carried out all of our analyses using three different methods, denoted herein as ‘Average Weight’, ‘Time Period’ and ‘Bonomi’;^112^ the principles behind each are fully detailed in the ESI† section ‘Metadynamics Re-weighting Schemes’. Qualitatively, there was overall good agreement in the properties of the system (AuBP1 internal conformation and modes of residue–surface contact) predicted by the three re-weighting methods, giving us confidence in our overall conclusions. Data presented herein were calculated using the ‘Average Weight’ method only; results derived using the ‘Time Period’ and ‘Bonomi’ procedures are given in the ESI (see Table S3 and Fig. S8, S10 and S13†). A brief summary of the ‘Average weight’ approach is provided in the Methods.

Overall, our conformational analysis of AuBP1 revealed few clear distinctions between the three aqueous gold interfaces. The conformational ensembles of the peptide were predicted to be approximately the same for all three interfaces, with a favoring of polyproline II and random coil character. These conformations were also predominant in bulk solution, in agreement with experiment.^23,28^ The variations between the three interfacial REST-metaD simulations seen from our analysis were too small to reasonably support the notion that differential surface-induced folding was the mechanism responsible for the binding selectivity predicted here (full exposition of this analysis is given in the ESI† section ‘Peptide Conformation’). Therefore, given the internal configuration of AuBP1 was considered as approximately equivalent at the three aqueous interfaces, we suggest that differences in the contact made between each residue and the surface gold atoms, must play a significant role. To investigate this, we calculated the unbiased probability with which each residue in AuBP1 was in contact with each Au surface. Direct adsorption to the Au surface was defined using residue-specific distance-based cut-offs derived from the van der Waals extent of relevant side-chain atoms (ESI† section ‘Analysis: Binding Residues’).

**Fig. 4:**
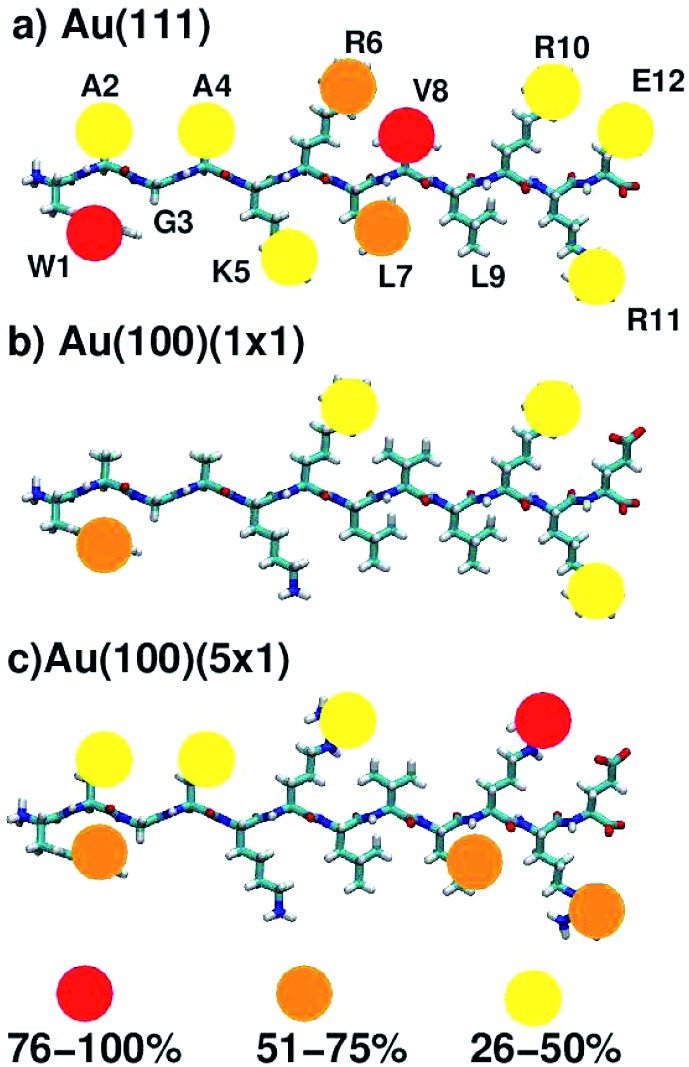
Schematic indicating residues that mediate peptide–surface contact at the aqueous (a) Au(111), (b) Au(100)(1 × 1) and (c) Au(100)(5 × 1) interfaces. The strength of the contact is indicated by color. See text for further details.

On average, 5.9, 4.6 and 2.0 residues were predicted to be simultaneously in direct contact with the surface at the aqueous Au(111), Au(100)(5 × 1) and Au(100)(1 × 1) interfaces, respectively. This trend in the number of contact residues, with Au(111) > Au(100)(5 × 1) > Au(100)(1 × 1), correlates with the peptide adsorption free energy at the three interfaces (Table 1). In addition, when the binding propensity of the twelve residues in AuBP1 is considered and ranked in turn, it is not only the *number* of anchoring points between AuBP1 and the Au surface, but also their quality (see Fig. 4, and S13 in the ESI†), that increases concurrently with the binding affinity of the peptide as a whole. By ‘quality’ we refer to the propensity of individual residues in AuBP1 for a given surface. Residues were classed as ‘strong’ binders if the probability of the residue being in direct contact with the gold surface was in the range 75–100%, ‘significant’ 51–75% and ‘moderate’ 26–50%.

As illustrated in Fig. 4, adsorption to Au(111), the interface with the strongest binding affinity, was mediated by ten out of the twelve residues (2 ‘strong’, 2 ‘significant’ and 6 ‘moderate’). In contrast, only seven contact residues were identified for the Au(100)(5 × 1) interface (1 ‘strong’, 3 ‘significant’ and 3 ‘moderate’). The Au(100)(1 × 1) interface supported the lowest number of contacts overall, four in total, none of which were strong (1 ‘significant’, 3 ‘moderate’). While, in some instances, each re-weighting scheme yielded an assignment of different *absolute* numbers of residues in each category, the *trend* across the three facets was qualitatively consistent across the three re-weighting schemes used. The significantly greater degree of direct contact made between AuBP1 and the Au(111) and Au(100)(5 × 1) interfaces, compared to Au(100)(1 × 1), further supports our hypothesis of there being two distinct modes of peptide–gold adsorption: the first mediated by direct contact with surface gold atoms, and the second mediated by a layer of interfacial water^74^ (see Fig. 3). Thermodynamic data calculated here indicate that the second, solvent-separated adsorption mode is quantitatively weaker than the first.

Exemplar histograms of the residue–surface separation (generated for values of the CV corresponding the peptide-bound state), shown in Fig. S14 of the ESI,† revealed that binding residues (those that consistently showed strong-to-significant contact across all three facets) in AuBP1 have a clear propensity to adsorb within the first interfacial water layer (‘direct contact’) on Au(111). On the Au(100)(1 × 1) surface, however, two adsorption modes are apparent, with the second corresponding to adsorption within the second interfacial water layer; this effect is particularly pronounced for Arg. To further underscore this, in Fig. S15 of the ESI† we show examples of typical binding configurations for each of the three facets, superimposed against the three-dimensional interfacial water density for each case. We also investigated the vertical interfacial water density profiles, calculated both with and without the presence of the adsorbed peptide; these data, however, did not reveal any significant changes in the spatial structuring of the solvent at the interface.

Further evidence of the influence of interfacial water structuring on the mode of peptide binding on the facets is provided *via* calculation of the average number of first layer interfacial water molecules displaced by the peptide when in the adsorbed state (associated with values of the CV corresponding to the bottom of the free energy well). We probed this for our two extreme cases, the Au(111) and Au(100)(× 1) facets. We found that AuBP1 adsorption on the Au(111) facet released an average of 35 ± 10 water molecules, compared with 5 ± 8 for AuBP1 adsorbed on Au(100)(1 × 1). Therefore, our data indicate that AuBP1 adsorption on the Au(100) facet features a strong element of mediation *via* the first interfacial solvation layer.

The data for all three interfaces reveal a common feature, in that all showed at least moderate binding of the three Arg residues (R6, R10, R11) and the single aromatic Trp residue (W1). At the aqueous Au(100)(1 × 1) interface, where binding was predicted to be weakest, AuBP1 was effectively adsorbed to the surface by these three Arg residues and Trp only. This tallies with expectations generated from our previous simulation studies comparing the adsorption of Arg (in amino acid form) at the Au(100)(1 × 1) and Au(100)(5 × 1) interfaces^74^ that indicated that Arg is a moderate binder on the Au(100)(1 × 1) surface, but showed greater and more persistent contact with the Au(100)(5 × 1) surface. In marked contrast with the other two interfaces, none of the remaining residues were adsorbed for more than 25% of the REST-metaD trajectory, indicating a lack of supporting interactions along the length of the peptide chain at Au(100)(1 × 1). At the other extreme, AuBP1 at the Au(111) interface showed strong and persistent contact along the entire length of the chain, with Arg and Trp featuring among the strongest contacts. This is in broad agreement with both our previously published REST simulations^13,50^ and REMD simulations^82^ of the AuBP1/Au(111) aqueous interface.

Our previous studies, using the FFs applied in this work, have reported predictions of the adsorption free energy of all twenty naturally-occurring amino acids at the aqueous Au(111) interface,^13^ indicating that both Arg and Trp are predicted to be strong-binding, at least in amino acid form. However, due to the interplay between sequence, conformation(s) and binding propensity, the affinity of a peptide cannot be simply considered as an additive sum of amino acid binding preferences. For this reason, our previous studies have indicated that some residues (in the peptide) can show a variability in the degree of surface contact, depending on their position in the chain and their immediate environment, even when the calculated free energy of binding of the corresponding amino acid is strong.^50^ In addition, contacts with at least ‘moderate’ strength were seen for residues with much less propensity on the amino acid level to interact,^13,50^ such as Ala, Leu, Val and Glu.

The extent of AuBP1 binding at the aqueous Au(100)(5 × 1) interface lies between the two extremes of Au(111) and Au(100)(1 × 1). Interestingly, two of the three Arg contacts were classified as being stronger than ‘moderate’, compared with one out of three for the Au(111) case. Given that the guanidinium group is predicted to lie flat in its direct-adsorbed configuration, it is plausible that the rumpled Au(100)(5 × 1) surface facilitates an approximately equivalent degree of contact with the surface compared with the atomistically-flat Au(111) counterpart. On the other hand, the affinity of Trp, with its larger planar side-chain functional group, appeared to be slightly greater for the atomically flat Au(111) surface than the rumpled Au(100)(5 × 1) interface. One possible explanation for these contrasting behaviors is the aspect ratio of the guanidinium group of Arg compared with the indole group of Trp; the long aspect ratio of the indole perhaps prevented good contact across the corrugations of the Au(100)(5 × 1) surface, while there was no such hindrance on the flat Au(111) surface. Conversely, the length-scales of the guanidinium group and the corrugations on the Au(100)(5 × 1) surface may be more similar, and therefore the differences in the degree of contact on the two surfaces may be relatively smaller. However, there was insufficient evidence to suggest whether the bulkier, branched, aliphatic side-chains of Leu (L7, L9) and Val (V8), both of which could feasibly have a greater degree of surface area contact with an undulating surface than an atomically flat one, contributed more to AuBP1 adsorption on Au(100)(5 × 1) than Au(111). We hypothesise that the matching of the relative length-scales of the residue side-group and the undulations on the Au(100)(5 × 1) surface may play a role in conferring a binding preference to Au(100)(5 × 1) over Au(111), such that both Arg, and the smaller aromatic residues (Tyr and Phe) may bind comparably well at Au(111) and Au(100)(5 × 1), while larger groups (*e.g.* Trp) may not. The relationship between residue shape and their relative binding propensity to the aqueous Au(111) and Au(100)(5 × 1) interfaces could be tested in the future. With such information it may be possible to design Au(100)(5 × 1) selective peptide sequences.

## Conclusions

Using Replica Exchange with Solute Tempering combined with Metadynamics simulations of the Au-binding peptide AuBP1 adsorbed at three aqueous gold interfaces, we presented predictions of facet-dependent *free* energies for peptide–gold adsorption. These data suggest that AuBP1 adsorption to the Au(111) interface is thermodynamically favored over Au(100), in aqueous solution. The binding affinity of the peptide to the three aqueous interfaces was clearly correlated with both decreasing structure within the first layer of interfacial adsorbed water molecules^73,74^ and with an increasing number and quality of residue–gold contacts, but not with differences in the peptide conformational ensemble. Our findings strongly indicate that interfacial water structuring was critical to conferring selective peptide adsorption at the three different crystallographic planes of gold. Two distinct modes of binding existed: the first involved direct contact between the peptide and atoms in the gold surface, like that for the Au(111) and Au(100)(5 × 1) interfaces; and the second was mediated by a tightly bound layer of interfacial water molecules between gold and the peptide, seen for the Au(100)(1 × 1) case. Although our conclusions hold irrespective of the reconstructed status of the Au(100) surface, our calculations predict that the binding affinity of AuBP1 for the Au(100)(1 × 1) interface is quantitatively weaker than that for Au(100)(5 × 1). Therefore the exact structure of Au(100) facets featured by gold nanoparticles in solution could impact on the resulting nanoparticle morphology, if such nanoparticles were used to seed gold precipitation in the presence of peptides such as AuBP1.
