# Supplementary material for: Facet selectivity in gold binding peptides: exploiting interfacial water structure
Source: Chem Sci. 2015 Jun 23;6(9):5204–14. doi: 10.1039/c5sc00399g (PMC5669244; doi:10.1039/c5sc00399g)
Supplement: Supplementary file 1 [file SC-006-C5SC00399G-s001.pdf]

Electronic Supplementary Material (ESI) for Chemical Science.
This journal is © The Royal Society of Chemistry 2015

Electronic Supplementary Information: Facet selectivity in gold binding peptides: exploiting interfacial water structure†

Louise B. Wright[a], J. Pablo Palafox-Hernandez[b], P. Mark Rodger*[a,c], Stefano Corni*[d] and Tiffany R. Walsh*[b]

a Dept. of Chemistry, University of Warwick, Coventry, CV4 7AL, U.K.

b Institute for Frontier Materials, Deakin University, Geelong, 3216, VIC, Australia; tiffany.walsh@deakin.edu.au

c Centre for Scientific Computing, University of Warwick, Coventry, CV4 7AL, U.K.; p.m.rodger@warwick.ac.uk

d Centro S3 CNR Istituto Nanoscienze, Modena, Italy; stefano.corni@nano.cnr.it

Contents

Computational Methods

Figure S1: Exemplar mobilities of representative replicas (0, 5, 10, 15) through effective temperature space.

Figure S2: Symmetrized final free energy profiles of AuBP1 adsorption at the aqueous a) Au(111), b) Au(100)(1×1)and c) Au(100)(5×1)interfaces.

Metadynamics Re-weighting Schemes

Figure S3: Histograms of the relative frequency with which the biased CV (peptide *com*–gold distance) was sampled for systematically establishing equilibration times.

Figure S4: Free Energy Profiles G_{f1} and G_{f5} of AuBP1 adsorbed at the three aqueous aqueous interfaces.

Analysis

Table S1: Estimation of surface energies for each of the aqueous gold interfaces in the presence and absence of AuBP1.

Figure S5: Boundaries in ϕ/ψ space marking the principal regions in a Ramachandran plot.

Figure S6: Reference structures *a-i* used to cluster conformations of AuBP1 at the aqueous Au(111), Au(100)(1×1) and Au(100)(5×1) interfaces.

Table S2: Amino acid functional group gold distances used as cut-off to define direct gold adsorption at the aqueous Au(111), Au(100)(1×1) and Au(100)(5×1) interfaces.

Peptide Conformation

Figure S7: Secondary structure assignments of AuBP1 when **a)** free in solution, and adsorbed at the three aqueous interfaces, using the 'Average Weighting' scheme.

Figure S8: Secondary structure assignments of AuBP1 when **a)** free in solution, and adsorbed at the three aqueous interfaces, using the 'Time Period' and 'Bonomi' methods.

Figure S9: Percentage population of clusters for AuBP1 in solution and adsorbed at the three aqueous interfaces, using the ‘Average Weighting’ scheme.

Figure S10: Percentage population of clusters for AuBP1 in solution and adsorbed at the three aqueous interfaces, using the ‘Time Period’ and ‘Bonomi’ methods.

Table S3: Percentage of ‘globular’ and ‘extended’ structures predicted using the different re-weighting methods.

Convergence of Metadynamics Simulations

Figure S11: Time evolution of the free energy of adsorption of AuBP1.

Figure S12: Evolution of the free energy profile of AuBP1–gold adsorption with metadynamics simulation time for the three interfaces.

Figure S13: Degree of residue–surface contact, indicated by colored circles, for AuBP1–gold adsorption at the three interfaces, using two re-weighting approaches.

Table S4: Summary of *in vacuo* binding energies of amino acid analogues on the three Au facets.

Figure S14: Histogram of residue–surface distance for exemplar residues at the three aqueous interfaces, generated from CV values corresponding to the surface-adsorbed state of the peptide.

Figure S15: Exemplar configurations of AuBP1 adsorbed at the three aqueous interfaces, superimposed against the three-dimensional interfacial water density.

References

Computational Methods

System Setup

Both Molecular Dynamics (MD) and metadynamics (metaD) simulations of AuBP1 (WAGAKRLVLRRE)[1], performed here were carried out using the software package GROMACS 4.5.5[2] and an in-house customized version of PLUMED 1.3[3]. Four different systems were considered: the isolated peptide in solution and, the peptide adsorbed at the aqueous Au(111), Au(100)(1$\times$1) and Au(100)(5$\times$1) interfaces. The first comprised the AuBP1 peptide solvated by 6605 TIP3P water molecules in a cubic simulation cell of length 58.28Å. Orthorhombic cells of dimensions $58.60 \times 60.90 \times 67.60$ Å^3, $58.60 \times 58.60 \times 67.60$ Å^3 and $58.60 \times 58.60 \times 76.51$ Å^3 were used for the interfacial simulations carried out at the Au(111), Au(100)(1$\times$1) and Au(100)(5$\times$1) surfaces, respectively. These comprised the AuBP1 peptide, a gold slab (5/5/9 layers thick) and 6605/6540/6355 TIP3P water molecules. The protonation state of the peptide at pH 7 was modelled, with 3 Cl^{-1} counterions added to balance the charge. The depth of water between the top surface of the slab and the bottom surface of its image was comparable in all three sets of interfacial simulations, being $\sim$60 Å.

In all simulations, CHARMM22*[4,5] was chosen to model the peptide, while the modified TIP3P[6,7] potential, with which the bio-organic CHARMM FF has been harmonized, was used to represent water. Bond lengths within water were constrained by the SETTLE algorithm[8]. Peptide-gold interactions at each of the different aqueous gold interfaces were described by GolP-CHARMM[9,10]. Simulations were carried out in the canonical (constant number, volume and temperature, NVT) ensemble with the temperature maintained at 300 K using a Nosé-Hoover thermostat[11,12] with a relaxation time of 0.2 ps. In all cases, the cell dimension in the z dimension was adjusted prior to simulation to ensure that the density of liquid water far from both the substrate and peptide was consistent with that for TIP3P water simulated under the same conditions in an isobaric-isothermal (constant number, pressure and temperature, NPT) ensemble at a pressure of 1 bar. Newton's equations of motion were solved using the Leapfrog algorithm[13] with an integration time-step of 1 fs; co-ordinates were saved every 1 ps. Particle mesh Ewald electrostatic summation was truncated at 11 Å, while a force-switched cut-off starting at 9 Å and ending at 10 Å was used for LJ non-bonded interactions. Cubic interpolation was used with 0.12 nm Fourier spacing and an Ewald tolerance of 10^{-6}.

REST simulations

All REST[14–16] simulations were set up in an analogous manner to our previous work[17,18]. Briefly, a total of 16 replicas were used to span an effective temperature range of 300-433 K (300.00, 305.35, 310.89, 317.25, 323.88, 331.57, 339.88, 349.11, 358.07, 367.37, 380.98, 389.80, 398.22, 406.85, 419.97, 433.00 K). Each replica was initially populated with AuBP1 present in a different conformation; peptide structures were constructed by hand to feature common folded backbone secondary structural motifs. All REST simulations featured the same set of internal-peptide and peptide-gold (orientation with respect to and distance from the gold surface) starting conformations. The peptide was initially close to the top surface of the gold slab in 13 out of the 16 replicas, in the centre of the cell in 2 replicas, and close to the nearest periodic image of the bottom face in the one remaining replica. Before initiating REST, starting configurations were equilibrated by a short MD simulation, of duration 1 ns, at their target potentials. No exchange moves were attempted and no bias was added to the collective variable during this time. In the subsequent production simulations, exchanges between neighbouring replicas were attempted every 1 ps.

The CV chosen for the interfacial REST-metaD runs was the position of the centre of mass (*com*) of the peptide in the z dimension (the direction normal to the gold surface). Gaussians of width, σ=0.1 Å, and height, w=0.1 kJ mol^{-1}, were added every 0.5 ps along the direction of the CV. REST-metaD simulations were run for 100 ns, making a total of 1.6 μs of dynamics per interface. All replicas were mobile throughout the whole of effective temperature space (See Figure S1b for trajectories along the effective temperature ladder of representative replicas in each simulation). The two REST MD simulations; one of AuBP1 in solution and the other of the peptide at the aqueous Au(111) interface; were carried out for comparative purposes and were run for 15 ns only.

Free energy extraction and error analysis

In the limit of an infinite metaD simulation ($t \to \infty$), the bias added during a metadynamics simulation approaches the negative of the free energy of the system, $V(X,t) \to -G(X,t)$, where V, G and X are the metadynamics bias added, the free energy of the system and its co-ordinates, respectively. The symmetrical nature of our simulation set-up means that two estimates of the binding affinity for each crystallographic plane could be attained per run; adsorption to the top face of the Au slab (ΔG_{ads_t}) and adsorption to the underside of the periodic neighboring slab (ΔG_{ads_b}) respectively[19] (referred to herein as the bottom face). Using the definition of Schneider and Colombi Ciacchi[20], ΔG_{ads_t} and ΔG_{ads_b} were estimated using:

$$\Delta G_{ads_t}(t_f) = -k_B T\, ln\left(\frac{c_{ads_t}}{c_{bulk}}\right) \tag{1}$$

$$\Delta G_{ads_b}(t_f) = -k_B T\, ln\left(\frac{c_{ads_b}}{c_{bulk}}\right) \tag{2}$$

where c_{ads_t} is the concentration of adsorbed peptide at the top face, c_{ads_b} is the concentration of the adsorbed peptide at the bottom face, and c_{bulk} is the peptide concentration in the bulk. These concentrations are calculated as:

$$c_{ads_t} = \frac{1}{z_0 - z_{min}} \int_{z_{min}}^{z_0} exp[-G(X,t_f)/k_B T]\, dX \tag{3}$$

$$c_{bulk} = \frac{1}{z_1 - z_0} \int_{z_0}^{z_1} exp[-G(X,t_f)/k_B T]\, dX \tag{4}$$

$$c_{ads_b} = \frac{1}{z_{max} - z_1} \int_{z_1}^{z_{max}} exp[-G(X,t_f)/k_B T]\, dX \tag{5}$$

where z_0 and z_1 indicate the values of the CV for which the peptide is considered to be in the 'bulk' solution (*i.e.* the peptide was defined as not adsorbed for $z_0 < z < z_1$, and was defined as adsorbed for all other values of z; see Figure S2). z_{min} is the z coordinate of the top (upper side) of the gold slab, and correspondingly z_{max} is the z coordinate bottom surface of the underside of slab as its periodic image. T is the temperature and t_f = 100 ns, the duration of the simulations performed here. Specifically, z_0 was defined using the final symmetrized free energy profiles of each system (see Figure S2) to be the minimum value of the CV for which $G(X,t_f) > -4$ kJ mol^{-1}; z_1 was then assigned the same distance from the bottom surface of the periodic image of the slab. Herein, we quote our calculated binding free energies as ΔG_{ads}, the mean of ΔG_{ads_t} and ΔG_{ads_b}. Its associated error was defined as half the difference between ΔG_{ads_t} and ΔG_{ads_b}. By

defining ΔG_{ads} in this manner the difference between the standard state of the experimental system and our model are minimized[21].

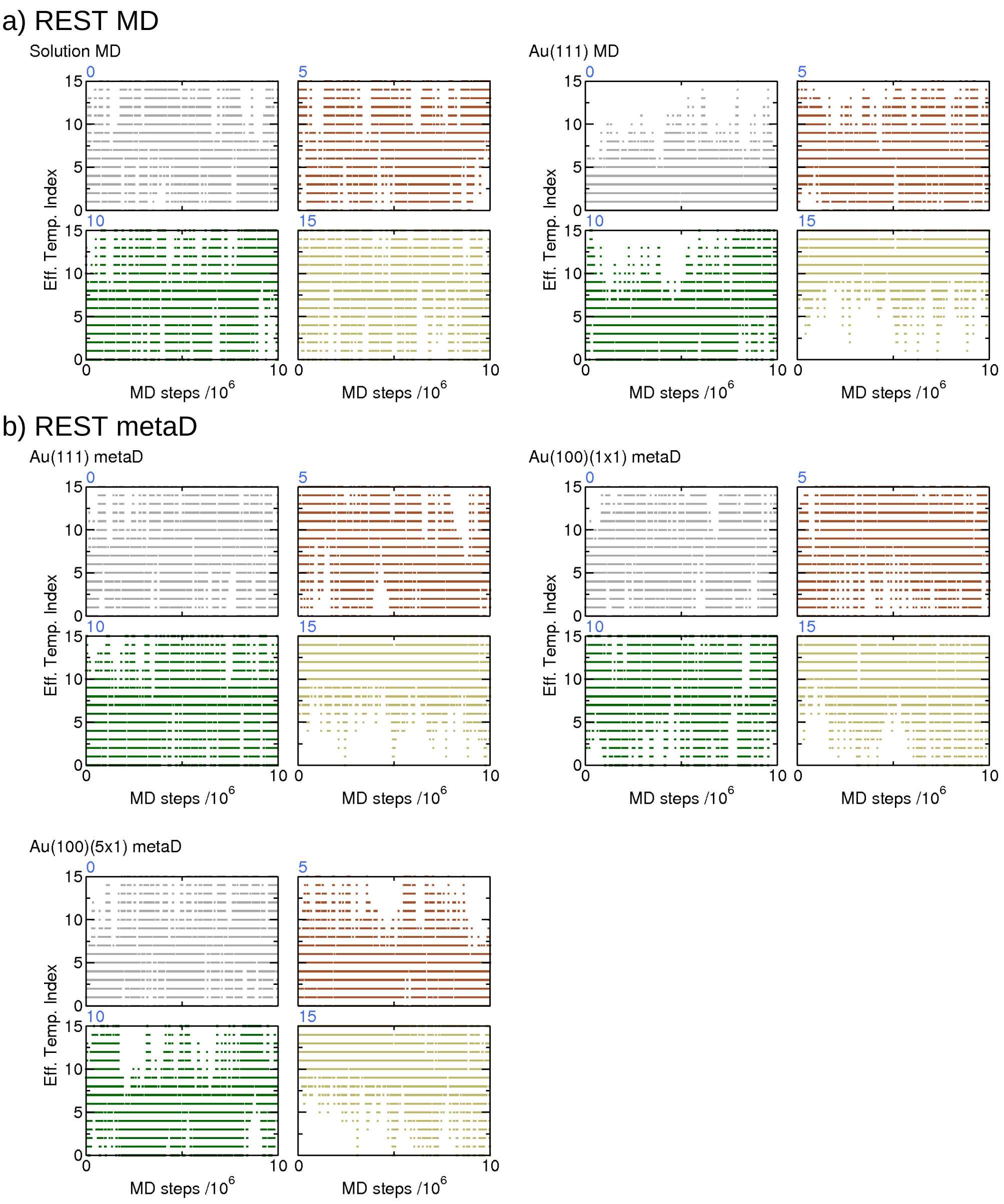

Figure S1: Trajectories of representative replicas (0, 5, 10, 15) through effective temperature space during **a)** REST MD simulations (of AuBP1 in solution and at the aqueous Au(111) interface) and during **b)** REST metaD simulations (at the aqueous Au(111), Au(100)(1×1) and Au(100)(5×1) interfaces). Data shown for first 10 ns of all simulations only.

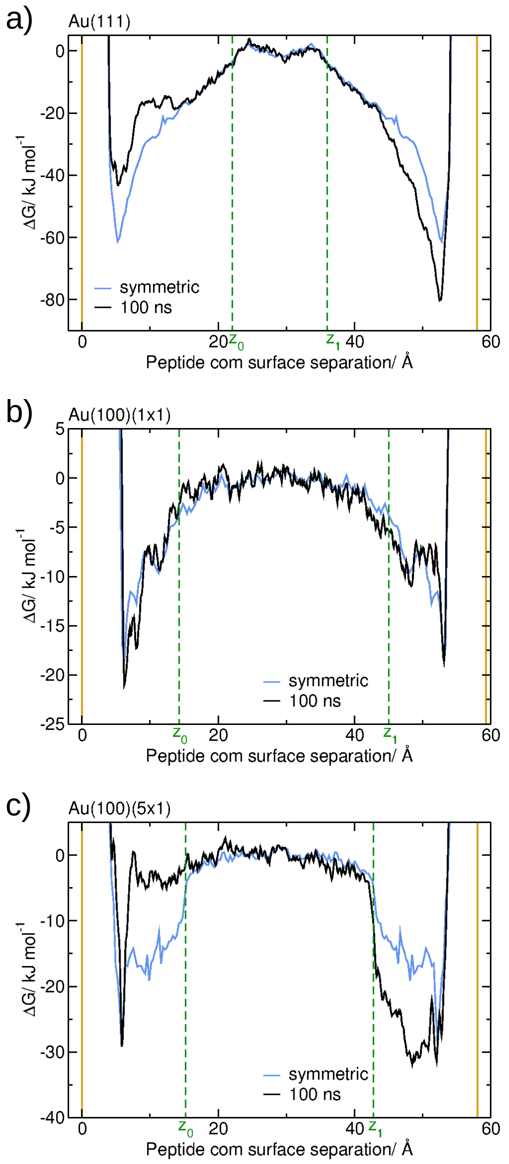

Figure S2: Symmetrized final free energy profiles of AuBP1 adsorption at the aqueous a) Au(111), b) Au(100)(1×1)and c) Au(100)(5×1)interfaces. z_0 and z_1 mark the extent of the 'adsorbed' zone for the peptide defined in Equations (2-4) in the SI.

Metadynamics Re-weighting Schemes

While in recent years metadynamics (metaD) has become a more frequently employed technique within the realm of molecular simulation, its use thus far reported in the literature has mainly been focused on either accelerating rare event sampling (for example simulating the crystallization of ice[22]) or for calculating free energy landscapes[20,23,24]. Analysis of the structural data generated by such simulations has largely been neglected due to the extreme challenge in relating the biased metaD trajectory to the structural properties of the system in the ensemble of interest. Specifically, the bias (V) added to a collective variable (CV), s, during a metaD simulation evolves with time according to:

$$V(X,t) = w\Sigma_{t'<t}\Pi_i exp\left[-\frac{(s_i(X(t)) - s_i(X(t')))^2}{2\sigma_i^2}\right] \tag{6}$$

where $X(t)$ are the co-ordinates of the system at time t.

Schemes for re-weighting basic[25] and well-tempered[26] metaD trajectories have been reported in the literature. The first, derived by Tiana, requires knowledge of x (the biased CV), y (the unbiased CV of interest), the potential energy of the entire system, U, and metaD bias, V, each time the bias is updated and at all time intervals in between[25]. Like others[26], we found the algorithm to be numerically unstable due to large fluctuations in system potential energy and therefore have discounted its use in the present study.

Of the few very recent studies where detailed structural analysis of metaD trajectories has been carried out[24,27,28], all employed the well-tempered metaD method[29] and the re-weighting procedure derived by Bonomi *et al.*[26]. Here, however, the basic, not well-tempered, metaD method was used. Therefore, in addition to the Bonomi re-weighting method, we have sought to find alternative methods of re-weighting our trajectories in order to sample the unbiased ensemble of interest. In total three schemes—labelled 'Average Weight', 'Time Period' and 'Bonomi'—were used; each is outlined below. Qualitatively, there is good agreement in the properties of the system (AuBP1 structure and modes of gold binding) predicted by the three re-weighting methods, giving us confidence in the overall conclusions drawn. Data presented in the main text was calculated using the 'Average Weight' method only; that derived using the 'Time Period' and 'Bonomi' procedures is given throughout the SI (Table S3, and Figures S8, S10, and S13).

'Average Weight'

The 'Average weight' method, the simplest of the three re-weighting schemes used here, weights all structures with a given peptide *com*–surface distance equally. The scheme is similar in concept to that used by Branduardi *et al.*[30]. Frames from the metaD trajectory were assigned to a grid along the biased CV, s. The weight of a frame, $W(t)$ is then given by:

$$W(t) = exp(-G(s(t,t_f))/kT)/N(s) \tag{7}$$

where $W(t)$ is the weight given to a frame sampled at time t, $G(s,t_f)$ the symmetrized free energy profile of the system at the end ($t = t_f$) of the simulation (Figure S2), and $N(s)$ the total number of frames for which the peptide *com*–gold distance was s. The appropriateness of this method for re-weighting a metaD simulation depends on two assumptions: 1) degrees of freedom orthogonal to the biased CV are sampled extensively from the correct ensemble and 2) $G(s)$ is converged. The first assumption is general to all the re-weighting schemes discussed here. By using REST alongside metaD, sampling of different adsorbed

peptide conformations (the degrees of freedom, orthogonal to the biased CV, of most interest in this study) is enhanced.

As discussed in SI Section 'Convergence of metadynamics', the free energy surface (FES) for AuBP1 adsorption onto gold is still evolving in the final stages of all three interfacial trajectories (Figure S11, S12). In particular, due to the way the simulations were set up, the final FES for AuBP1 adsorption at the aqueous Au(111) and Au(100)(5$\times$1)interfaces are not symmetrical; this is not expected as the two surfaces presented by the gold slab to solution are identical. Therefore, to address the second assumption upon which this 'Average Weight' method is based, $G(s)$ employed in Eqn 7 was the symmetrized final free energy profile (Figure S2) for the interface in question. In addition, data for analysis (and determination of $N(s)$ in Eqn 7) was only taken from the metaD trajectories after a period of equilibration. The length of the equilibration period (70 ns Au(111), 50 ns Au(100)(1$\times$1), 50 ns Au(100)(5$\times$1)) was determined by histogramming the biased CV at different times during the simulations (Figure S3). Ideally, when converged each point along this CV should be equally sampled. Sampling in the central region, midway between the two gold surfaces, was chosen as the metric against which convergence was measured here. Specifically, the period of equilibration was the shortest time for which the standard deviation of sampling in this central region of the cell was 8%, or less, than the mean.

'Time period'

A naive method for re-weighting a metaD simulation might be to weight each frame by the bias added to the CV at the time when the frame was sampled, $V(s(t))$ (Eqn 6). However, $V(s(t))$ continues to grow during a simulation, therefore inherently giving more importance to frames sampled in the later stages of trajectory. In the 'Time Period' re-weighting scheme, we propose to calculate the unbiased probability of an observable, *Obs*, on small intervals of time (t'=1–n); structures are weighted and normalized within each time segment using the average bias potential for the interval. The unbiased ensemble average value of *Obs*, $< Obs >$, is:

$$< Obs >= \frac{1}{\sum_{t'=1}^{n} R_{t'}} \left(R_1 \frac{\int_0^{t_1} \int Obs(X(t'),t')\bar{B}(X)\mathrm{d}X\mathrm{d}t'}{\int_0^{t_1} \int \bar{B}(X)\mathrm{d}X\mathrm{d}t'} + R_2 \frac{\int_{t_1}^{t_2} \int Obs(X(t'),t')\bar{B}(X)\,\mathrm{d}X\mathrm{d}t'}{\int_{t_1}^{t_2} \int \bar{B}(X)\,\mathrm{d}X\mathrm{d}t'} + ... \right) \tag{8}$$

where:

$$B(s(X(t))) = exp\left(\frac{V(X(s(t)))}{kT}\right) \tag{9}$$

In this scheme we used a time interval of 1 ns. Specifically the work flow was as follows:

1. Calculate the re-weighted average of the observable within each segment of time $Obs_{t'}$, t=t_1 to t=t_2. To do this, the weight given to a frame at time = t was set equal to $exp\{V_{t2}(s(X(t)),t)/kT\}$ (*i.e.* the bias added to the CV being sampled at time t determined using the bias potential generated at the end of the time segment, time t_2). $Obs_{t'}$ was normalized in each time segment to counterbalance the fact that the total bias, V($s(X(t)),t$), grows continuously during a simulation.

2. The contribution of the average value of the observable over an individual time period, $Obs_{t'}$, to the overall trajectory-averaged value, $< Obs >$, was determined by the range of the CV being sampled in the time interval, t'. Time periods during which the simulation samples the CV close to a free energy

minimum extensively should be given more importance than those during which it primarily visits higher energy regions. The weight given to each time period thus requires prior knowledge of the free energy profile of the system along the direction of the biased CV, $G(s(X))$. The average weight given to a time interval, R(t'), was:

$$R(t') = \frac{\sum_{t=t_1}^{t=t_2} exp(-G(s(X(t)))/kT)}{t_2 - t_1} \tag{10}$$

As discussed in SI Section 'Convergence of Metadynamics', and above for the 'Average Weight' method, for complex systems such as those considered in this study, both the slow diffusion of the peptide and convergence in sampling along directions on the FES orthogonal to the biased CV, introduce errors into the free energy profile, $G(s)$, derived at the end of the simulation.

3. However, within the 'Time Period' re-weighting method, it is possible to systematically improve the free energy profile in a self-consistent manner. Using the weights derived from iteration i, the CV biased in a simulation can be treated as an observable. Its unbiased probability distribution, P($s(X)$) can therefore be calculated from the simulation data. Inverting P($s(X)$) according to the standard relationship:

$$G = -kT ln(P(X)) \tag{11}$$

generates $G_{f_{i+1}}(s(X))$, an improved free energy profile which can be fed-back into step 2, above.

4. After 5 rounds of iterations, the free energy profiles of the three systems investigated in this work–AuBP1 adsorption at the aqueous Au(111), Au(100)(1×1) and Au(100)(5×1) interfaces–were observed to converge. The 5th generation profile, $G_{f5}(X)$, was used in the re-weighting of all data presented herein for the 'Time Period' method (Figure S4).

'Bonomi'

Derived by Bonomi *et al.*, this method was designed for re-weighting CVs orthogonal to those to which the metaD bias was added, for well-tempered[29] metaD simulations[26]. As described in detail elsewhere[26], assuming the metaD bias, V($s(X(t)$), evolves adiabatically, Parrinello and *co-workers* suggested that the rate at which the biased distribution of an observable changes, $P_{biased}(Obs, t+\Delta t)$, can be related to the rate of change of V, $\dot{V}$, by the approximate expression:

$$P_{biased}(Obs, t+\Delta t) = exp\{-(\dot{V}(s(Obs), t) - <\dot{V}(s,t)>)\Delta t/kT\} P_{biased}(Obs, t) \tag{12}$$

From $P_{biased}(Obs, t)$ and V($s(X(t)), t$), the unbiased probability distribution $P_{unbiased}$ can be recovered. The algorithm needed to carry out this re-weighting procedure is distributed as part of the PLUMED software package[3] and can be implemented when post-processing a well-tempered metaD simulation. This scheme has become an established method for re-weighting *well-tempered* metaD simulations in the literature[24,27,28]. Following the precedent set by Deighan and Pfaendtner, in their parallel tempering well-tempered interfacial simulations of peptides LKα14 and LKβ15 adsorbed on self assembled monolayers[28], we have performed our 'Bonomi' re-weighting only on frames in which the peptide was adsorbed to the gold surface. Adsorbed states were selected based on the free energy profile (Figure S12); specifically frames in which the peptide *com*–gold distance was in the range 4-12 Å Au(111), 6-13 Å Au(100)(1x1) and 4-14 Å

Au(100)(5x1). This is in contrast to both the ‘Average Weight’ and ‘Time Period’ methods, where analysis included data from *all* frames; using these two methods, adsorbed states are inherently exponentially weighted much greater than those in solution and hence dominate the results.

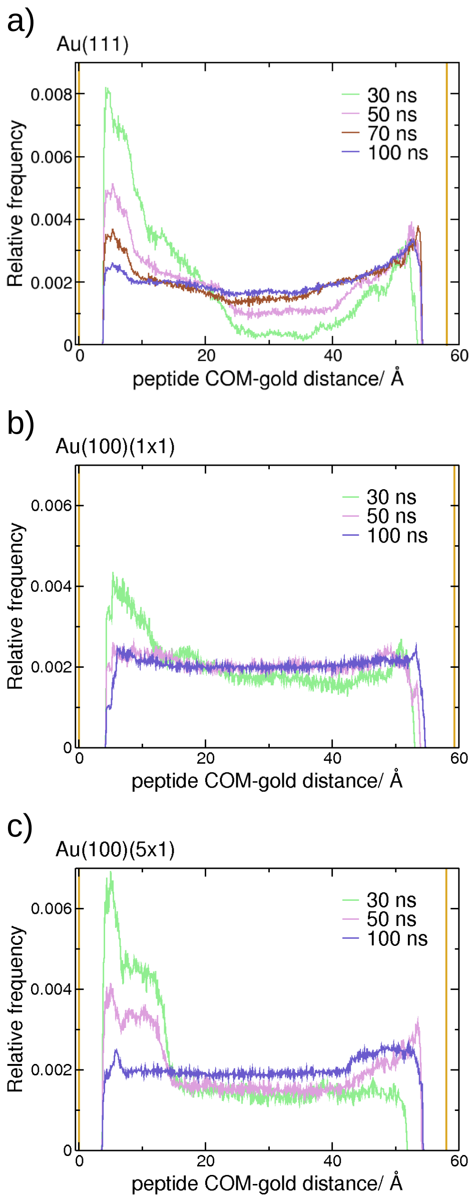

Figure S3: Histograms of the relative frequency with which the biased CV (peptide *com*–gold distance) was sampled after 30, 50 and 100 ns for the a) Au(111), b) Au(100)(1×1) and c) Au(100)(5×1) surfaces.

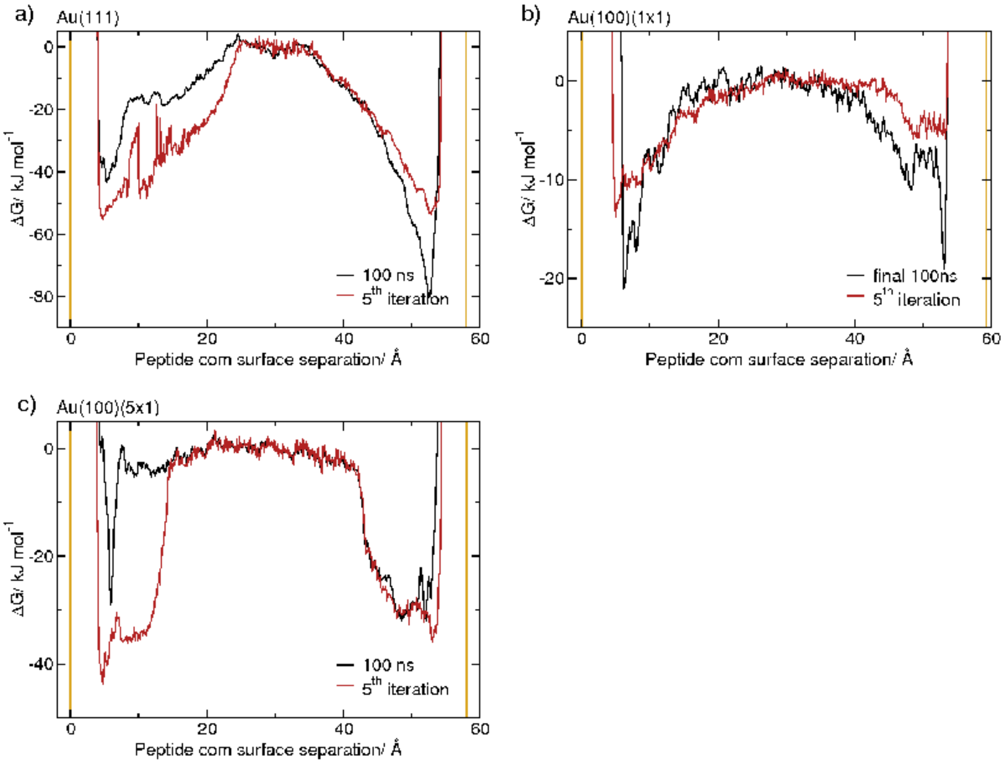

Figure S4: Free Energy Profiles G_{f1} and G_{f5} of AuBP1 adsorbed at the aqueous a) Au(111), b) Au(100)(1$\times$1) and c) Au(100)(5$\times$1) interfaces.

Analysis

Polycrystalline Surfaces

In this work we have estimated the composition of polycrystalline gold surfaces using Wulff constructions of AuNP of increasing size. Here we assume that AuNP (and in the limit of infinite size, polycrystalline gold) feature only Au(111) and Au(100) facets. Thermodynamically stable nanoparticle (NP) sizes and shapes can be predicted from the relative surface energies (γ) of their featured facets, according to Wulff's theorem[31]. Using a combination of experimental data available in the literature AuNP morphology was predicted using the 'Solid of Wulff Open Source' software[32] seeded with cubo-octahedral NP.

Heinz *et al.* reported $\gamma_{SL}^{[111]}$ to be 1.41-1.47 $\mathrm{J\,m^{-2}}$ [33] from experimental data[34] and the assumption that the water wetting coefficient for gold is 1.0. Exact values for $\gamma_{SV}^{[100]}$ are not available in the literature. Flueli and Borel, however, estimated the ratio $\gamma_{SV}^{[100]}/\gamma_{SV}^{[111]}$ to be $\sim$1.05[35]. Santos and Schmickler reported a difference of 1.9 $\mathrm{kJ\,mol^{-1}}$/(1×1) between the native and reconstructed Au(100) surfaces[36]. Thus, in our model we have taken $\gamma_{SL}^{[111]} = 1.47\,\mathrm{J\,m^{-2}}$, $\gamma_{SL}^{[100]native} = 1.54\,\mathrm{J\,m^{-2}}$ and $\gamma_{SL}^{[100]recon} = 1.50\,\mathrm{J\,m^{-2}}$ (Table S1). Like Heinz[33], we assume a water wetting coefficient of 1.0 in all cases to estimate γ_{SL} from γ_{SV}.

In good agreement with Table S1, previous detailed analysis, combining both first-principles calculations and high-resolution transition electron microscopy, report 60-70% of AuNP surface area to be Au(111) for NP of 15 nm diameter or greater at 300 K[37,38].

The affinity of AuBP1 for polycrystalline gold depends critically on peptide solution concentration. In the limit of single molecule adsorption to a polycrystalline surface, such as that modelled in our simulations, the binding affinity of the peptide will be dominated by adsorption to the most favorable surface sites only– in this case Au(111) facets. Only when these sites become saturated will adsorption to other surfaces become significant. Hence we present two estimates of AuBP1 adsorption free energy to polycrystalline gold, $\Delta G_{polycryst}$: first, at low peptide surface densities, $\Delta G_{polycryst} = \Delta G_{Au(111)}$; and second, in the limit of monolayer saturation, $\Delta G_{polycryst}$ was estimated as a weighted average ΔG_{111} and ΔG_{100} (Table 1, main text) using the percentage surface areas in Table S1. It is noted that slight differences exist between our work and experiment, due to the definition of the standard state. However, Wei and Latour previously demonstrated that this difference has negligible impact on peptide binding affinities[21].

Structural Analysis

Analysis of the simulations reported herein was oriented to help answer two key questions: 1) how does the structure of AuBP1 differ when adsorbed to one crystallographic plane of gold compared to another and 2) is there evidence for energetic and/or spatial selectivity in adsorption between the aqueous Au(111) and Au(100) interfaces. Unless otherwise stated, the entire 100 ns REST metaD trajectory of the reference replica (the replica in which the effective and actual simulation temperatures are identical) was analysed in all three cases–Au(111), Au(100)(1×1) and Au(100)(5×1). It is recognised that both adsorbed and desorbed states of AuBP1 are sampled in these metaD simulations. The primary aim of this work was to investigate the former (surface-bound conformations) only. This can be achieved by re-weighting the bias added to the CV connecting the two states in a suitable way (SI Section 'Metadynamics Re-weighting Schemes'). The advantage of this method is that it circumvents the need for an arbitrarily chosen cut-off to be used to mark the transition from the peptide being adsorbed, to free in solution. Analysis of the two REST MD simulations, where presented, was performed on the final 5 ns of each run only.

A combination of secondary structural analysis, based on Ramachandran plots, and structure clustering was used to probe the potential for AuBP1 to fold in a differential manner on adsorption to Au(111) and Au(100).

Ramachandran Analysis

The secondary structural characteristics of AuBP1 were determined by the re-weighted probability with which different regions of ϕ/ψ-phase space were sampled during each trajectory. Cut-off boundaries for common folded peptide motifs are depicted in Figure S5.

Clustering Analysis

In this work we propose it is not appropriate to use the Daura cluster algorithm[39] for structure cluster analysis of the biased REST-metaD trajectories. Briefly, each structure within the pool of structures identified over the entire trajectory has a different weight, determined by the REST-metaD bias added to the simulation at the time at which the structure was sampled. These different weights must be accounted for, *both* when identifying reference structures and when assigning structures to clusters.

An alternative strategy for grouping together similar conformations of AuBP1 sampled during a simulation is to use a pre-defined set of reference structures. Unlike Deighan and Pfaendtner[28], we identified our reference structures from two REST **MD** simulations (*i.e.* not REST-metaD) of AuBP1. Specifically, the Daura algorithm[39] was used to cluster structures sampled in the last 5 ns of these REST MD simulations of AuBP1 in solution and at the Au(111) interface. Only data from the unbiased, reference replica was used. A root mean squared deviation of peptide backbone position cut-off of 3 Å was employed. Across the two simulations, the centroid structures of 8 clusters–S2, S3, S4, S6 (A5), A1 (S1), A2 (S5), A3 and A4 (where Ax and Sx are the xth most populated clusters of the Au(111) and solution runs respectively)–were distinct (by which we mean that the RMSD in peptide backbone atoms between any pair was greater than the cut-off used in the clustering). Hereafter, these structures were labelled *a*-*h* (Figure S6). Broadly, solution derived conformations *a*-*d* were more globular than the extended structures *e*-*h* found to dominate at the Au(111) interface in the REST MD simulation. A ninth distinct structure, *i*, was found only to feature significantly only after the more extensive REST-metaD simulations; it was also extended in nature.

Binding Residue Analysis

Residues which were involved in binding AuBP1 to an interface were identified using a set of criteria based on side-chain functional group surface separations. The maximum distance for which a residue could physically be interpreted as being in direct contact with the gold atoms in the upper-most layer of each surface was used to determine cut-offs for binding[40]. This distance was defined as the Au-X (X=side-chain functional group heavy atom as stated in Table S2) van der Waals equilibrium separation plus 1 Å. The binding propensity of a residue was classed as one of three categories based on its percentage likelihood of being adsorbed to an interface: 26-50% ‘moderate’, 51-75% ‘significant’ and 76-100% ‘strong’.

	γ_{SL}/ J m^{-2}	SA nat/ %	SA rec/ %
Au(111)	1.47	66.6	62.7
Au(100)(1×1)	1.54	33.4	n/a
Au(100)(5×1)	1.50	n/a	37.3

Table S1: Surface energies (γ_{SL}) for each of the aqueous gold interfaces and percentage of total AuNP surface area (SA) featuring Au(111) and Au(100) native (nat) or reconstructed (rec) facets.

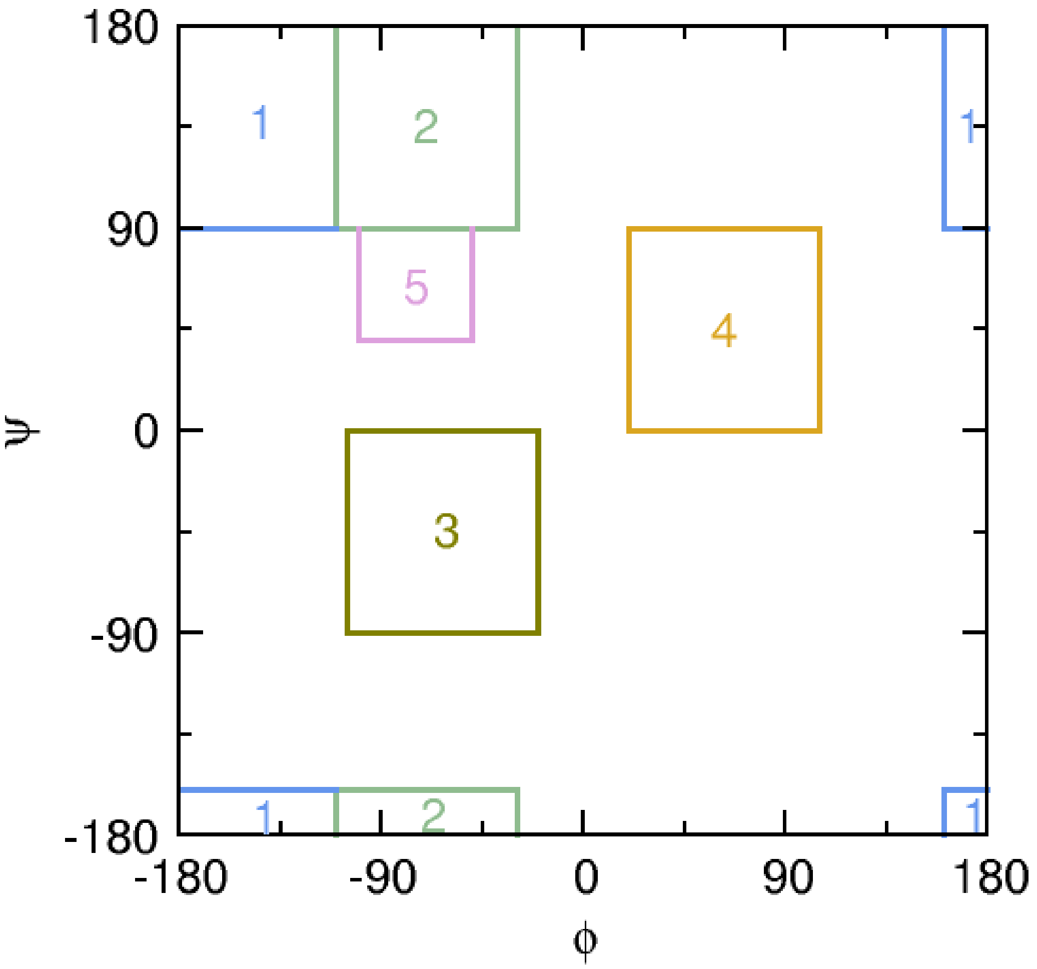

Figure S5: Boundaries in ϕ/ψ space marking the principal regions in a Ramachandran plot (1 β, 2 PPII, 3 α, 4 α_L and 5 γ_L) for analysis of AuBP1 structure.

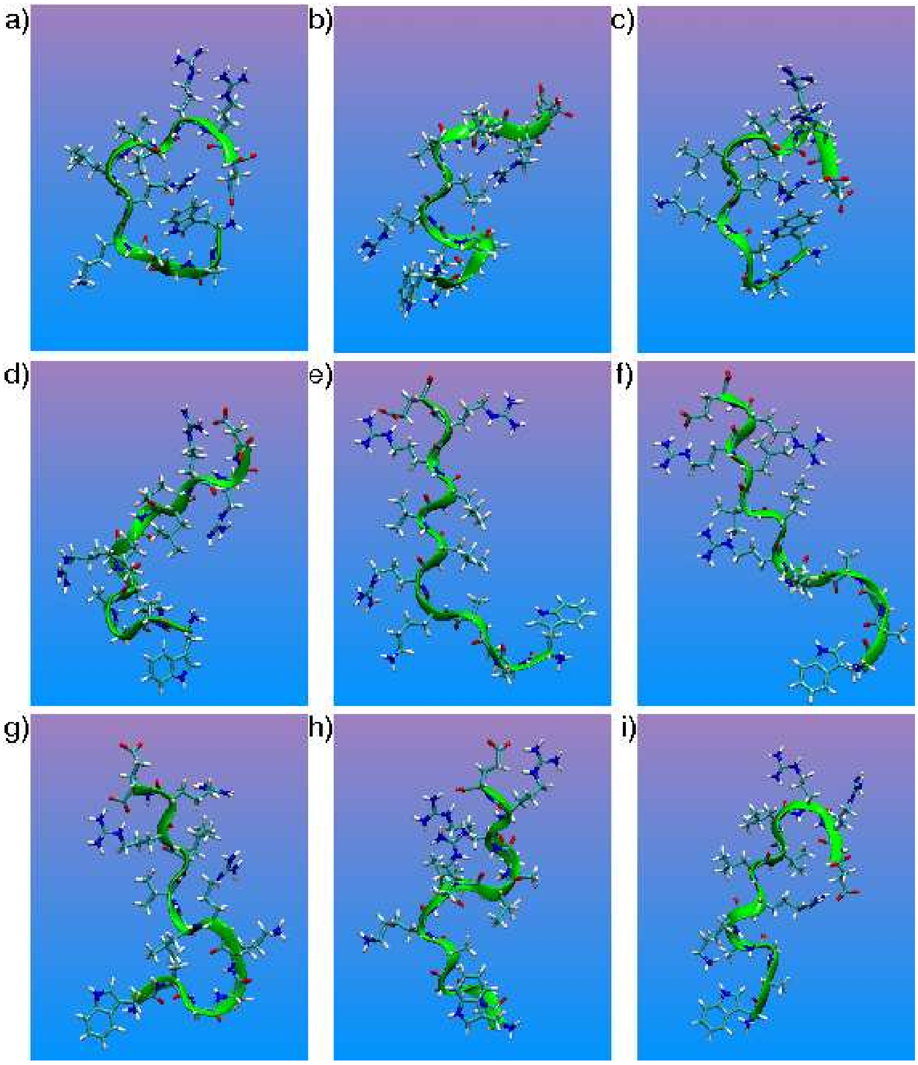

Figure S6: Reference structures a-i used to cluster conformations of AuBP1 at the aqueous Au(111), Au(100)(1×1) and Au(100)(5×1) interfaces identified during the REST metaD simulations.

	funct. group	$\mathrm{Dir}_{Au(111)}$ / Å	$\mathrm{Dir}_{Au(100)(1\times1)}$ / Å	$\mathrm{Dir}_{Au(100)(5\times1)}$ / Å
ALA	C_β	4.7	4.8	4.6
ARG	guanidinium group	4.5	4.6	4.4
GLU	$\mathrm{CO}\mathbf{O}^-$	4.1	4.4	4.2
GLY	$C_\alpha\mathbf{H}_2$	3.1	3.2	3.0
LEU	C_δ	4.7	4.8	4.6
LYS	-$\mathbf{N}\mathrm{H}_3^+$	4.5	4.6	4.4
TRP	phenyl ring	4.4	4.4	4.4
VAL	C_γ	4.7	4.8	4.6

Table S2: Amino-acid functional group gold distances used as cut-off to define direct gold adsorption at the aqueous Au(111), Au(100)(1×1) and Au(100)(5×1) interfaces. In the case of Arg and Trp, the *com* of the heavy atoms in the functional group described above was used in determining residue side-chain surface separation, whereas for Glu, Gly, Leu and Val it was the *closest* carboxylate oxygen atom, hydrogen or methyl carbon, respectively.

Peptide Conformation

Ramachandran plots

Peptide secondary structural analysis based on Ramachandran plots and structural clustering (see SI Section 'Analysis: Structural Analysis') were both used to investigate the possibility for AuBP1 to fold in a differential manner upon adsorption to each of the three facets. Each common secondary structure protein motif (*e.g.* α-helix, β-sheet, *etc*) is associated with a specific combination of the peptide backbone torsional angles, ϕ and ψ. The secondary structural characteristics of AuBP1 were therefore determined by the re-weighted probability with which different regions of ϕ/ψ space (as defined in Figure S5) were sampled during the REST-metaD simulation (see Figures S7 and S8). First, it is noteworthy to compare the predicted ensemble of structures adopted by AuBP1 in solution (*i.e.* in the absence of the Au surface) with the experimental data available[1,41]. The computational model (CHARMM22*/TIP3P) appears to be in good agreement with experiment; both experimental studies and our predictions revealed a propensity for AuBP1 to adopt structures with polyproline II (PPII) and random coil characteristics in solution (Figure S7a). Second, our simulations are suggestive of an environmental dependence in the secondary structural characteristics of AuBP1, although slight differences in the outcomes from our three re-weighting schemes prevents a definitive conclusion on the significance of this dependence. Data from both the 'Average Weight' (see Figure S7) and 'Time Period' (Figure S8, top) re-weighting schemes suggest a decrease in the α character of AuBP1 upon adsorption to gold; this trend is less pronounced for the 'Bonomi' scheme, especially in the case of adsorption to the Au(100)(1$\times$1)interface (Figure S8, bottom). Recent Circular Dichroism spectroscopy measurements, probing the ensemble of conformations adopted by AuBP1, also suggest that adsorption to Au surfaces can perturb the configuration of the peptide in the aqueous environment[41]. However, taking the results from all three re-weighting schemes into account, it is not possible to make a definitive statement about differences in the adsorbed conformation of AuBP1 between the three aqueous gold interfaces.

Clustering Results

Similar conclusions can be drawn from our structural clustering analysis of AuBP1 (Figures S9 and S10). The reference structures upon which our cluster analysis was based were identified from two REST MD simulations of AuBP1. Peptide structures were assigned to reference clusters with the smallest root mean squared deviation (RMSD) in backbone atom positions; unassigned structures were those for which the RMSD to all reference cluster structures was greater than 3 Å. Further details on the cluster analysis reported here can be found in SI Section 'Analysis: Structural Analysis'. In general, the unbiased population of those clusters with more 'extended' type centroid structures (labelled *e-i* in Figure S9 featured more prominently than those with 'globular' conformations (labelled *a-d* in Figure S9) when the peptide was adsorbed to any of the gold surfaces (Table S3), again suggesting a shift in AuBP1 conformation upon adsorption to the Au surface. While our data, particularly from the 'Average Weight' (Figure S9) and 'Time Period' (Figure S10, top) re-weighting schemes, also revealed differences in the relative populations of each AuBP1 cluster when adsorbed to each of the three aqueous interfaces, no clear trend emerged. Perhaps the only consistent surface-dependent distinction in the adsorbed structures was a reduced probability of the peptide adopting structures belonging to cluster '*e*' at the Au(100)(5$\times$1)interface; '*e*' was the most populated cluster for the Au(111)and Au(100)(1$\times$1)surfaces.

	Average Weight		Time Period		Bonomi	
	a-d	e-i	a-d	e-i	a-d	e-i
	%	%	%	%	%	%
Solution	48.6	40.0				
Au(111)	23.8	63.2	13.0	85.6	34.3	59.6
Au(100)(1x1)	22.0	76.0	19.3	76.5	31.6	55.5
Au(100)(5x1)	41.0	47.7	16.7	82.3	22.5	69.7

Table S3: Percentage of 'globular' (*a*-*d*) and 'extended' (*e*-*i*) structures predicted using the different re-weighting methods. Analysis does not account for the globular or extended nature of unassigned structures.

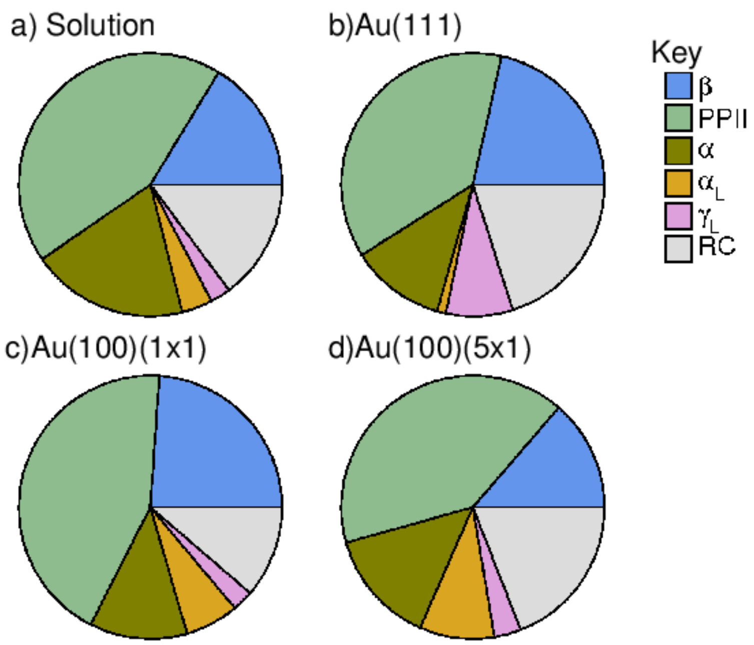

Figure S7: Secondary structure assignments of AuBP1 when **a)** free in solution, and adsorbed at the aqueous **b)** Au(111), **c)** Au(100)(1$\times$1)and **d)** Au(100)(5$\times$1)interfaces, using the 'Average Weighting' scheme.

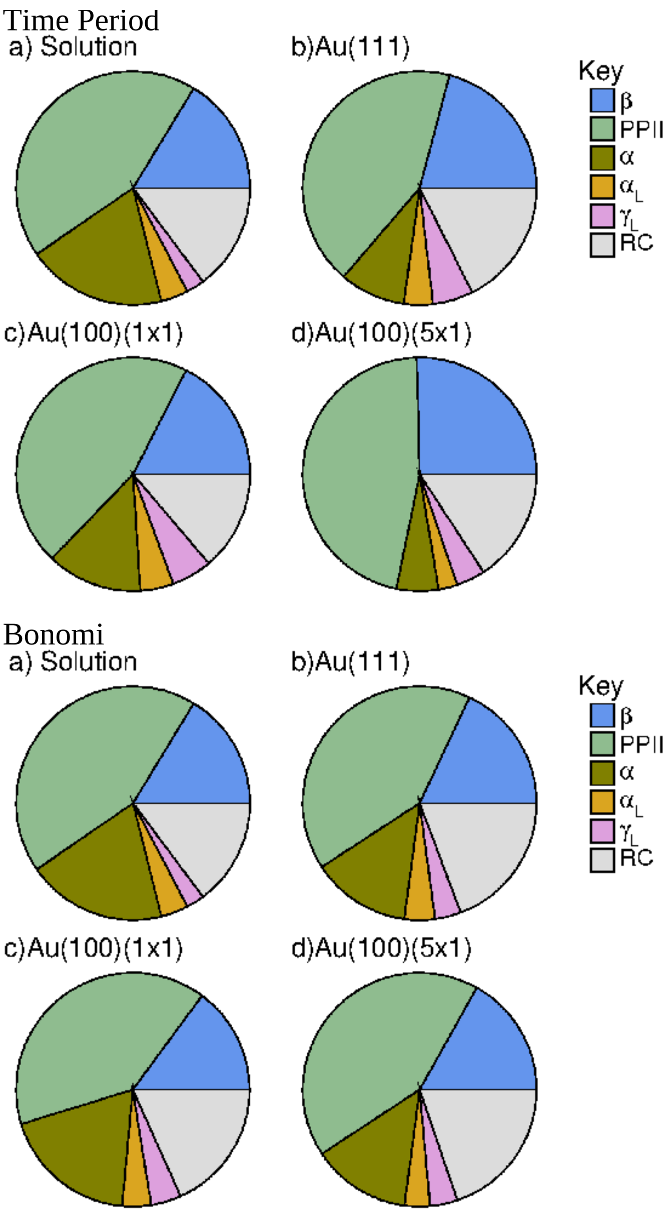

Figure S8: Secondary structural characteristics of AuBP1 when a) in solution and adsorbed at the aqueous b) Au(111), c) Au(100)(1$\times$1)and d) Au(100)(5$\times$1)interfaces. Top depicts data re-weighted using the 'Time Period' method and bottom that re-weighted using the 'Bonomi' method.

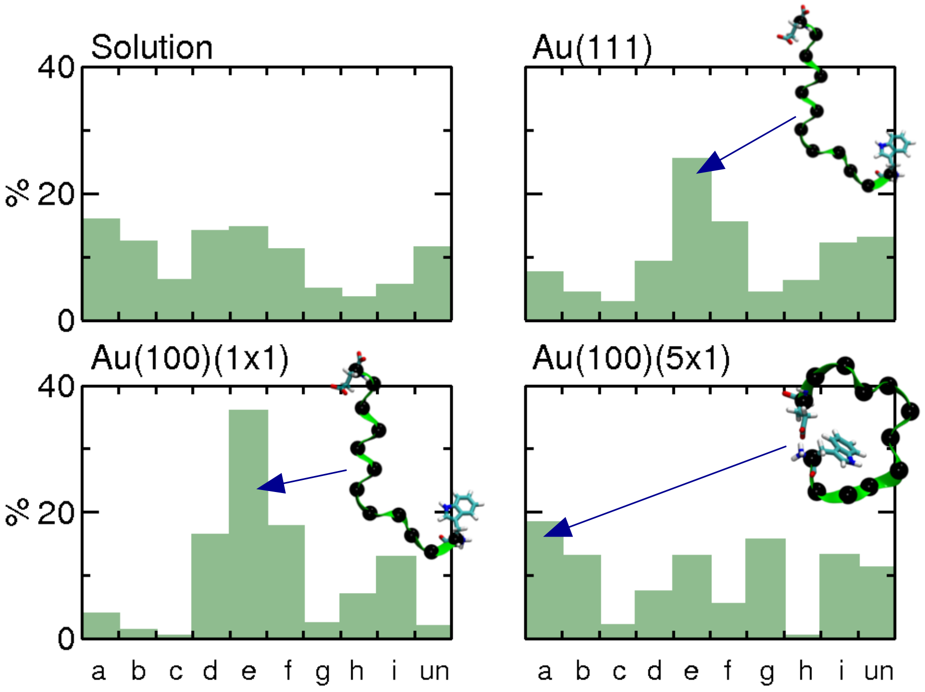

Figure S9: Percentage population of clusters *a-i* ('un' denotes unassigned structures) for AuBP1 in solution and adsorbed at the aqueous Au(111), Au(100)(1×1)and Au(100)(5×1)interfaces,using the 'Average Weighting' scheme.

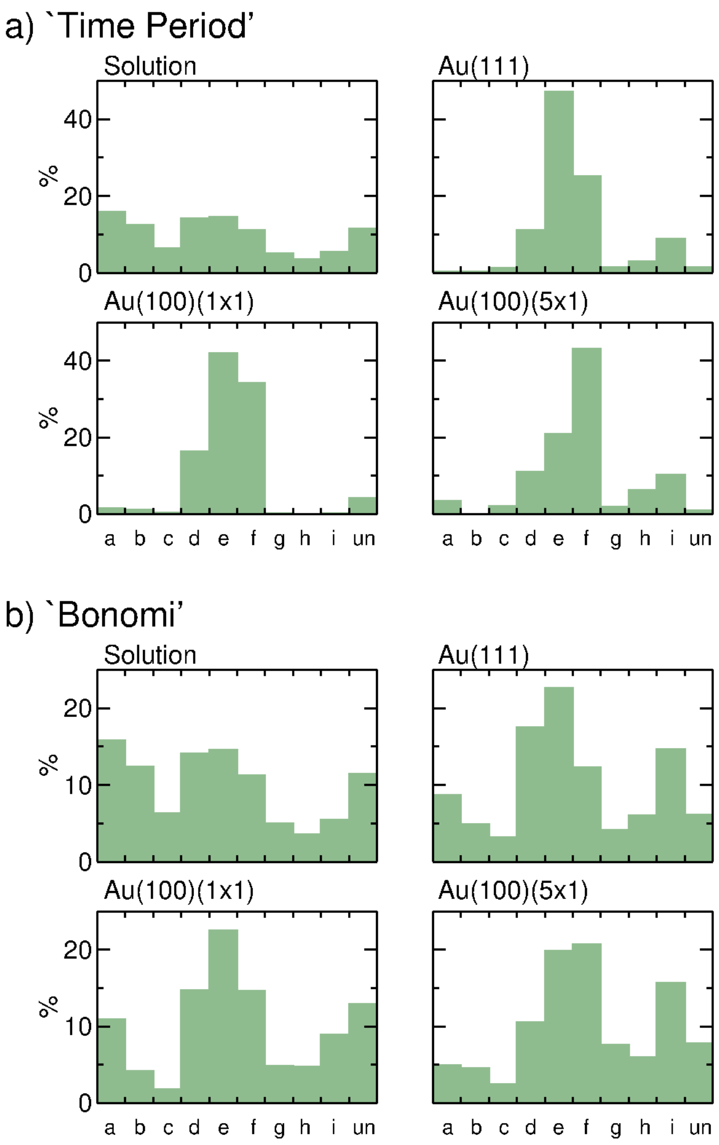

Figure S10: Percentage population of clusters a-i ('un' denotes unassigned structures) for AuBP1 in solution and adsorbed at the aqueous Au(111), Au(100)(1$\times$1)and Au(100)(5$\times$1)interfaces using data re-weighted using a) the 'Time Period' and b) the 'Bonomi' methods.

Convergence of Metadynamics

As highlighted by the recent paper by Jambeck and Lyubartsev[23], the inability to adequately sample all of conformational space orthogonal to a biased CV within the time period of a simulation hinders convergence of a metaD run. (By 'converged' we mean that the free energy profile is stationary within the limit of small fluctuations–on the order of magnitude of the height of the Gaussian hills added in the simulation.) Here we have used REST in combination with metaD specifically to enhance sampling of the peptide degrees of freedom orthogonal to the biased CV. In the case of the bio-interfacial systems modelled here, it is the inter-conversion between different modes of peptide adsorption, in particular, which could impede convergence. For instance, if the transition between two binding states of AuBP1 is only possible by the following mechanism–complete desorption, re-folding/orientation in solution and re-adsorption in an alternative manner–then the time scale for this process could be much slower than the rate at which the metaD bias is added. The free energy profile would, therefore, be observed to continue to evolve, rather than converge, in this instance.

One way to overcome this 'lack' of convergence is to use the well-tempered metaD method[29] and/or further biased CVs, connecting folded and unfolded peptide states. The latter, however, requires prior knowledge of favourable folded conformations for the particular peptide sequence. For example, in the interfacial simulations performed by Deighan and Pfaendtner, and, Meiβner *et al.*, peptides with known α-helical[24,28] and β-sheet[28] structure were modelled. AuBP1, on the other hand, is much less structurally defined, adopting an ensemble of different favourable conformations in solution (Figures S7 and S8).

The well-tempered metaD method is an attractive option for attaining a converged free energy profile within a computationally feasible timescale. Caution must be taken not to cease these simulations too early though. Specifically, in the later stages of a well-tempered metaD simulation, the bias added to the CV is extremely small such that the free energy profile inherently appears converged. However, sampling of conformational space orthogonal to the biased CV, which occurs over much longer timescales, may not be complete. In the work carried out here, metaD was primarily used alongside REST in order to overcome strong peptide-gold adsorption observed, in our preliminary REST MD simulations, to hinder exploration of peptide conformational space. Since the free energy of adsorption of AuBP1 at specific crystallographic planes of gold under aqueous conditions is currently not known experimentally, we sought only to estimate binding affinities. The basic metaD method, rather than well-tempered, method was therefore employed.

The symmetrical nature of the CV in the REST metaD simulations reported here means that two estimates of the binding affinity of AuBP1 for the crystallographic plane in question–the top and bottom surface of the gold slab and its periodic neighbour, respectively–can be attained per run. A simple estimate of the uncertainty in the binding free energy–adopted herein–at a given interface, is thus the difference between these two values. Others have calculated this error boundary as the maximum fluctuation of $\Delta G_{min}^{ads}(t)$ over the final stages of a metaD run, T_f, away from its average value, $< \Delta G_{min}^{ads} >_{T_f}$, taken over the same time period[20,24]. For AuBP1–gold adsorption the magnitude of the error estimated by the two different methods was comparable.

It is noted that even after 100 ns of simulation the shape of the adsorption free energy profile (Figure S12) and the magnitude of the free energy of adsorption (Figure S11) are still evolving, although to much less an extent than that observed in the initial stages of the runs. In addition to the difficulties in obtaining a 'converged' free energy profile for complex bio-interfacial systems in general mentioned above, convergence was further impeded here by the initial distribution of the peptide in replica space. Specifically, the peptide was clustered to one region of the CV (close to the upper surface of the gold slab) in the majority

of replicas (SI Section ‘Computational Methods: System Set-up’). Not only must the metaD bias be built up sufficiently in all replicas for the FES to be essentially flat, but sufficient time must have elapsed for a random walk on this landscape to disperse these clustered replicas across CV space, for the adsorption profiles to converge fully. This, in particular, leads to the asymmetry observed in the final free energy profiles of AuBP1 adsorption at the aqueous Au(111)and Au(100)(5$\times$1)interfaces.

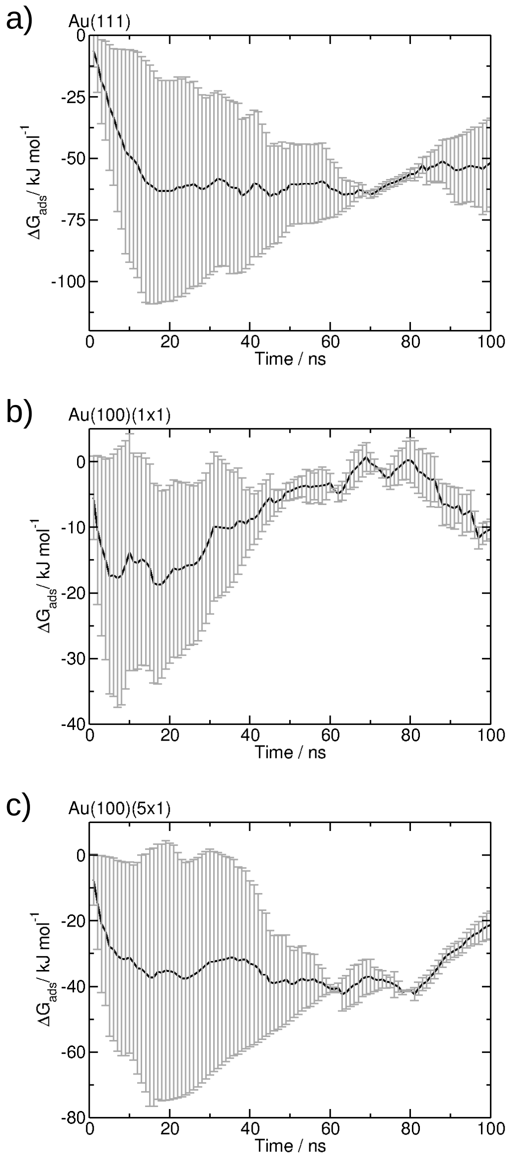

Figure S11: Time evolution of the free energy of adsorption of AuBP1 to a) Au(111), b) Au(100)(1$\times$1) and c) Au(100)(5$\times$1). Plotted in black is the average free energy change based on the peptide binding to the top and bottom surface of the gold slab; error bars represent the energetic differences between the two interfaces.

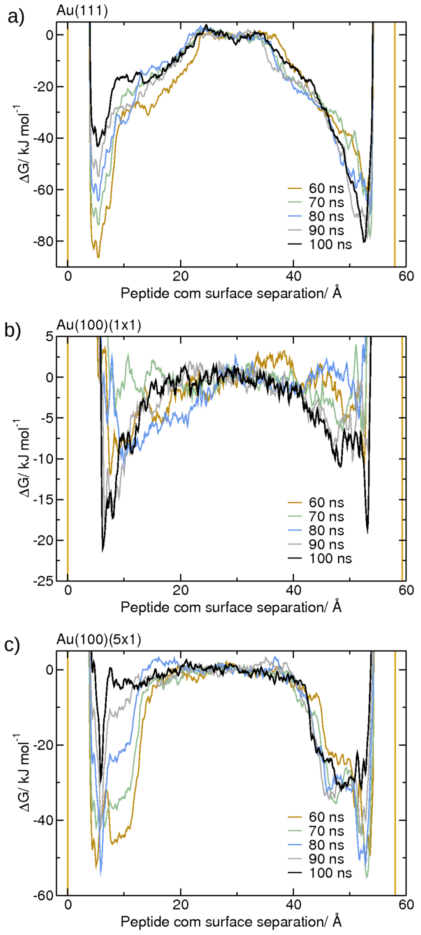

Figure S12: Evolution of the free energy profile of AuBP1–gold adsorption with metadynamics simulation time for the a) Au(111), b) Au(100)(1$\times$1)and c) Au(100)(5$\times$1)runs.

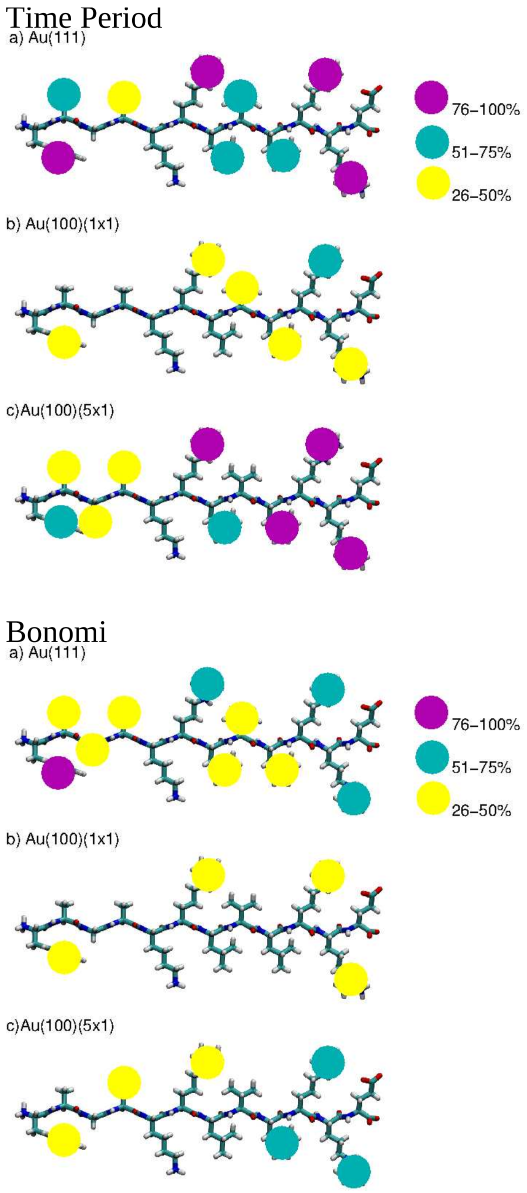

Figure S13: Schematic depicting residues which mediate AuBP1–gold adsorption at the aqueous a) Au(111), b) Au(100)(1$\times$1) and c) Au(100)(5$\times$1)interfaces. 'Strong' (percentage surface contact: 76-100%) binding residues are highlighted in magenta, 'significant' (51-75%) in cyan and 'moderate' (26-50%) in yellow. Top depicts data re-weighted using the 'Time Period' method and bottom that re-weighted using the 'Bonomi' method.

Adsorbate	Au(111)	Au(100)(5×1)	Au(100)(1×1)
water	-18.1	-21.8	-20.9
methane	-13.5	-17.5	-16.9
butane	-38.4	-45.4	-43.3
benzene	-60.8	-58.5	-58.4
methanol	-25.0	-30.5	-28.7
methanoic acid	-29.4	-36.5	-30.4
methylamide	-35.6	-39.8	-34.7
methanethiol	-46.5	-51.5	-51.6
methylamine	-54.8	-61.7	-60.8
imidazole	-54.5	-66.0	-67.0
diethylsulfide	-70.0	-91.2	-86.3

Table S4: Collation of *in vacuo* binding energies ($kJ\,mol^{-1}$) of amino acid analogues adsorbed onto the Au(111), Au(100)(5×1) and Au(100)(1×1) facets, calculated using the GolP-CHARMM force-field. Data taken from Wright *et al.*[9], Wright *et al.*[10] and Hughes *et al.*[42].

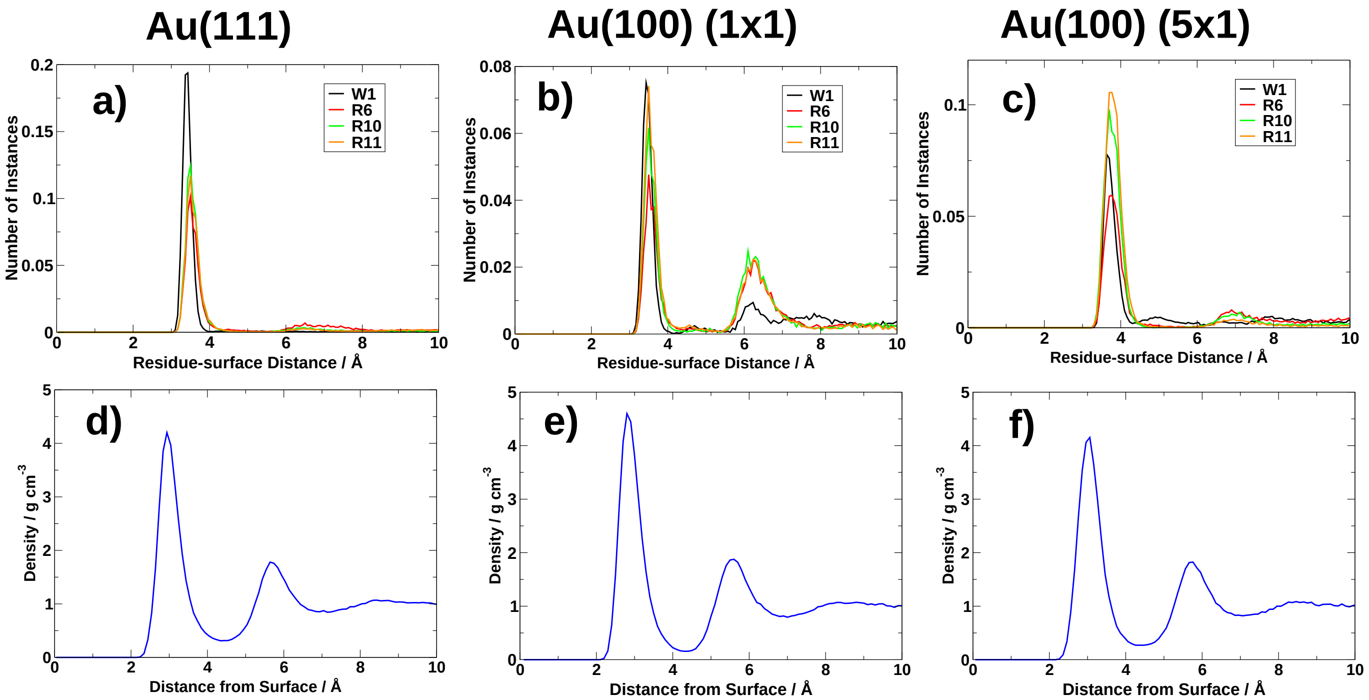

Figure S14: Histogram of the residue–surface distance for each ‘strong/significant’ binding residue that is common to all three facets: W1, R6, R10 and R11, for **a)** Au(111), **b)** Au(100)(1×1) and **c)** Au(100)(5×1). Vertical water density profiles are shown for comparison for **d)** Au(111), **e)** Au(100)(1×1) and **f)** Au(100)(5×1). The plots in **a), b)** and **c)** were generated for values of the CV corresponding to the peptide-adsorbed state.

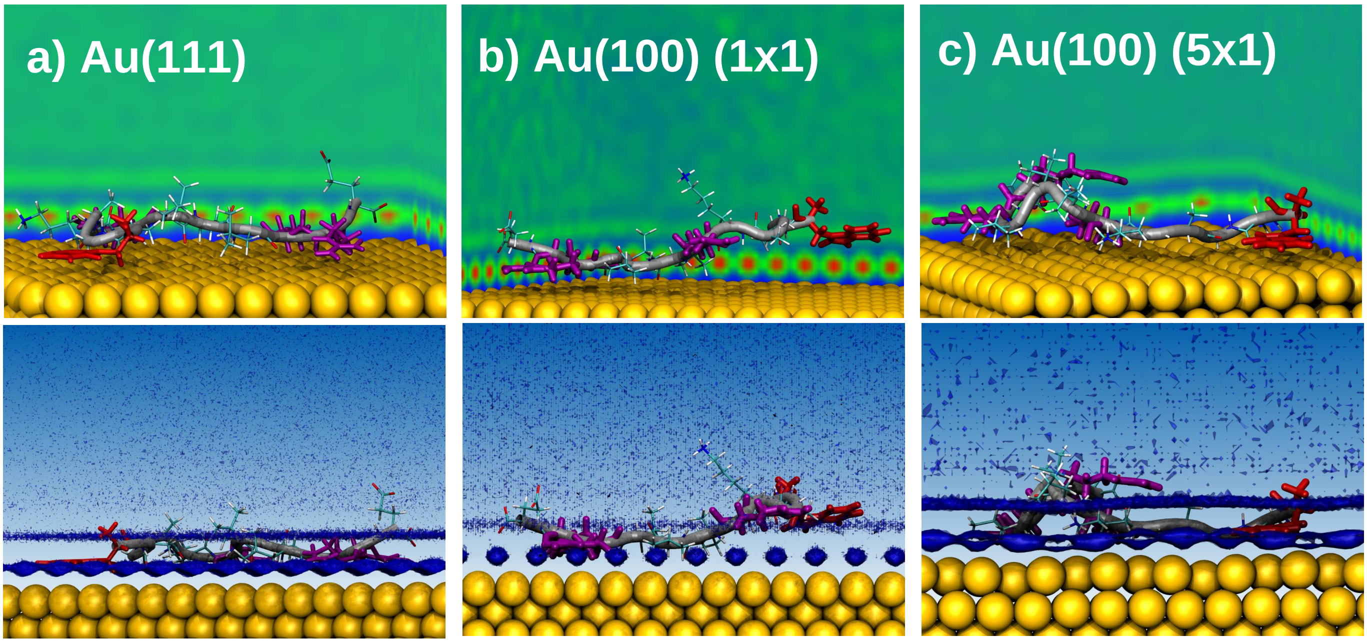

Figure S15: Exemplar configurations of AuBP1 adsorbed on **a)** Au(111), **b)** Au(100)(1$\times$1) and **c)** Au(100)(5$\times$1) surfaces, superimposed against the three-dimensional interfacial water density. Top and bottom images provide two different renderings of this water density. Differences in renderings between facets are due to adjustments in isosurface values (for visualisation purposes), and should not be interpreted quantitatively. The Trp residue is coloured red, the three Arg residues are coloured purple.

References

[1] M. Hnilova, E. E. Oren, U. O. S. Seker, B. R. Wilson, S. Collino, J. S. Evans, C. Tamerler and M. Sarikaya, *Langmuir* , 2008, **24**, 12440–12445.

[2] B. Hess, C. Kutzner, D. van der Spoel and E. Lindahl, *J. Chem. Theory Comp.*, 2008, **4**, 435–447.

[3] M. Bonomi, D. Branduardi, G. Bussi, C. Camilloni, D. Provasi, P. Rateri, D. Donadio, F. Marinelli, F. Pietrucci, R. A. Broglia and M. Paririnello, *Comp. Phys. Comm.*, 2009, **180**, 1961–1972.

[4] S. Piana, K. Lindorff-Larsen and D. E. Shaw, *Biophysical J.*, 2011, **100**, L47–L49.

[5] A. D. MacKerell, D. Bashford, M. Bellott, R. L. Dunbrack, J. D. Evanseck, M. J. Field, S. Fischer, J. Gao, H. Guo, S. Ha, D. Joseph-McCarthy, L. Kuchnir, K. Kuczera, F. T. K. Lau, C. Mattos, S. Michnick, T. Ngo, D. T. Nguyen, B. Prodhom, W. E. Reiher, B. Roux, M. Schlenkrich, J. C. Smith, R. Stote, J. Straub, M. Watanabe, J. Wiorkiewicz-Kuczera, D. Yin and M. Karplus, *J. Phys. Chem. B*, 1998, **102**, 3586.

[6] W. L. Jorgensen, J. Chandrasekhar, J. D. Madura, R. W. Impey and M. L. Klein, *J. Chem. Phys.* , 1983, **79**, 926–935.

[7] E. Neria, S. Fischer and M. Karplus, *J. Chem. Phys.* , 1996, **105**, 1902–1921.

[8] S. Miyamoto and P. A. Kollman, *J. Comp. Chem.* , 1992, **13**, 952.

[9] L. B. Wright, P. M. Rodger, S. Corni and T. R. Walsh, *J. Chem. Theory Comput.*, 2013, **9**, 1616–1630.

[10] L. B. Wright, P. M. Rodger, T. R. Walsh and S. Corni, *J. Phys. Chem. C*, 2013, **117**, 24292–24306.

[11] S. Nosé, *Mol. Phys.*, 1984, **52**, 255.

[12] W. G. Hoover, *Phys. Rev. A*, 1985, **31**, 1695.

[13] R. W. Hockney, S. P. Goel and J. Eastwood, *J. Comp. Phys.*, 1974, **14**, 148.

[14] S. L. C. Moors, S. Michielssens and A. Ceulemans, *J. Chem. Theory Comput.*, 2011, **7**, 231–237.

[15] T. Terakawa, T. Kameda and S. Takada, *J. Comput. Chem.*, 2011, **32**, 1228–1234.

[16] L. Wang, R. A. Friesner and B. J. Berne, *J. Phys. Chem. B*, 2011, **115**, 9431–9438.

[17] L. B. Wright and T. R. Walsh, *Phys. Chem. Chem. Phys.*, 2013, **15**, 4715–4726.

[18] Z. Tang, J. P. Palafox-Hernandez, W. Law, Z. E. Hughes, M. T. Swihart, P. N. Prasad, M. R. Knecht and T. R. Walsh, *ACS Nano*, 2013, **7**, 9632–9646.

[19] L. Bellucci and S. Corni, *J. Phys. Chem. C*, 2014, **118**, 11357.

[20] J. Schneider and L. C. Ciacchi, *J. Am. Chem. Soc.*, 2012, **134**, 2407–2413.

[21] Y. Wei and R. A. Latour, *Langmuir*, 2008, **24**, 6721–6729.

[22] D. Quigley and P. M. Rodger, *J. Chem. Phys.*, 2008, **128**, 154518.

[23] J. P. M. Jambeck and A. P. Lyubartsev, *J. Phys. Chem. Lett.*, 2013, **4**, 1781.

[24] R. H. Meissner, J. Schneider, P. Schiffels and L. C. Ciacchi, *Langmuir*, 2014, **30**, 3487–3494.

[25] G. Tiana, *Eur. Phys. J. B*, 2008, **63**, 235–238.

[26] M. Bonomi, A. Barducci and M. Parrinello, *J. Comput. Chem.*, 2009, **30**, 1615–1621.

[27] M. Deighan, M. Bonomi and J. Pfaendtner, *J. Chem. Theory Comput.*, 2012, **8**, 2189–2192.

[28] M. Deighan and J. Pfaendtner, *Langmuir*, 2013, **29**, 7999–8009.

[29] G. B. A. Barducci and M. Parrinello, *Phys. Rev. Lett.*, 2008, **100**, 020603.

[30] D. Branduardi, G. Bussi and M. Parrinello, *J. Chem. Theory Comput.*, 2012, **8**, 2247–2254.

[31] G. Wulff, *Z. Kristallogr.*, 1901, **34**, 449.

[32] D. Scopece, *J. Appl. Cryst.*, 2013, **46**, 811.

[33] H. Heinz, R. A. Vaia, B. L. Farmer and R. R. Naik, *J. Phys. Chem. C*, 2008, **112**, 17281–17290.

[34] W. R. Tyson and W. A. Miller, *Surf. Sci.*, 1977, **62**, 267.

[35] M. Flueli and J. P. Borel, *J. Crys. Growth*, 1988, **91**, 67.

[36] E. Santos and W. Schmickler, *Chem. Phys. Lett.*, 2004, **400**, 26.

[37] A. S. Barnard, X. M. Lin and L. A. Curtiss, *J. Phys. Chem. B*, 2005, **109**, 24465–24472.

[38] A. S. Barnard, N. P. Young, A. I. Kirkland, M. A. van Huis and H. Xu, *ACS Nano*, 2009, **3**, 1431–1436.

[39] X. Daura, K. Gademann, B. Jaun, D. Seebach, W. F. van Gunsteren and A. E. Mark, *Angew. Chem. Int. Ed.*, 1999, **38**, 236.

[40] S. Corni, M. Hnilova, C. Tamerler and M. Sarikaya, *J. Phys. Chem. C*, 2013, **117**, 16900–17003.

[41] Y. Li, Z. Tang, P. N. Prasad, M. R. Knecht and M. T. Swihart, *Nanoscale*, 2014, **6**, 3165–3172.

[42] Z. E. Hughes, L. B. Wright and T. R. Walsh, *Langmuir*, 2013, **29**, 13217.
